# The Genome Architecture of the Copepod *Eurytemora carolleeae —* the Highly Invasive Atlantic Clade of the *Eurytemora affinis* Species Complex

**DOI:** 10.1093/gpbjnl/qzae066

**Published:** 2024-09-27

**Authors:** Zhenyong Du, Gregory Gelembiuk, Wynne Moss, Andrew Tritt, Carol Eunmi Lee

**Affiliations:** Department of Integrative Biology, University of Wisconsin, Madison, WI 53706, USA; Department of Integrative Biology, University of Wisconsin, Madison, WI 53706, USA; Department of Integrative Biology, University of Wisconsin, Madison, WI 53706, USA; Department of Integrative Biology, University of Wisconsin, Madison, WI 53706, USA; Department of Integrative Biology, University of Wisconsin, Madison, WI 53706, USA

**Keywords:** Genome architecture, Arthropod, Crustacea, Invasion, Osmoregulation

## Abstract

Copepods are among the most abundant organisms on the planet and play critical functions in aquatic ecosystems. Among copepods, populations of the *Eurytemora affinis* species complex are numerically dominant in many coastal habitats and serve as food sources for major fisheries. Intriguingly, certain populations possess the unusual capacity to invade novel salinities on rapid time scales. Despite their ecological importance, high-quality genomic resources have been absent for calanoid copepods, limiting our ability to comprehensively dissect the genome architecture underlying the highly invasive and adaptive capacity of certain populations. Here, we present the first chromosome-level genome of a calanoid copepod, from the Atlantic clade (*Eurytemora carolleeae*) of the *E*. *affinis* species complex. This genome was assembled using high-coverage PacBio long-read and Hi-C sequences of an inbred line, generated through 30 generations of full-sib mating. This genome, consisting of 529.3 Mb (contig N50 = 4.2 Mb, scaffold N50 = 140.6 Mb), was anchored onto four chromosomes. Genome annotation predicted 20,262 protein-coding genes, of which ion transport-related gene families were substantially expanded based on comparative analyses of 12 additional arthropod genomes. Also, we found genome-wide signatures of historical gene body methylation of the ion transport-related genes and the significant clustering of these genes on each chromosome. This genome represents one of the most contiguous copepod genomes to date and is among the highest quality marine invertebrate genomes. As such, this genome provides an invaluable resource to help yield fundamental insights into the ability of this copepod to adapt to rapidly changing environments.

## Introduction

Copepods form the largest biomass of animals in the world’s oceans, and arguably on the planet [1]. Among estuarine and coastal copepods, the planktonic calanoid copepod *Eurytemora affinis* species complex is a dominant grazer throughout the Northern Hemisphere, forming an enormous biomass in estuaries and coastal habitats, with census sizes in the billions [2–4]. As such, this copepod represents a major food source for some of the world’s most important fisheries, such as herring, anchovy, flounder, and larval salmon [5–8].

The species complex *E*. *affinis* has been the focus of intense ecological and evolutionary interest because of its extraordinary ability to invade a wide range of salinities on ecological time scales [9–12]. This copepod has the rare ability for an invertebrate to cross salinity boundaries ranging from hypersaline to completely freshwater habitats [9,13–15]. Within a few decades, saline populations of this species complex have invaded freshwater habitats multiple times independently on three continents through human activity [9,16,17]. For instance, with the opening of the St. Lawrence Seaway, estuarine populations of the Atlantic clade of the *E*. *affinis* complex (also known as *Eurytemora carolleeae*) [18] were introduced into the North American Great Lakes from saline estuarine populations ∼ 65 years ago, starting with Lake Ontario in 1958 and reaching Lake Superior by 1972 [9,19]. Likewise, populations of the Gulf clade of the *E*. *affinis* complex spread rapidly from the Gulf of Mexico into inland freshwater reservoirs and lakes throughout the Mississippi drainage system over a time period of ∼ 80 years [9,17]. Additionally, a European *E*. *affinis* population survived the transformation of a saltwater bay in the Netherlands into freshwater lakes (IJsselmeer and Markemeer) over a period of six years [9,20]. These freshwater introductions by saline *E*. *affinis* complex populations were accompanied by the rapid evolution of freshwater tolerance, along with evolutionary changes in life history and ion regulatory function [13,14,21–25]. Natural selection experiments in the laboratory revealed that this rapid freshwater adaptation could occur within 6–10 generations [13,21,26].

Across these independent evolutionary transitions to novel salinities, natural selection has repeatedly acted on ion transport-related genes in the *E*. *affinis* complex populations [15,21,24]. Multiple prior studies have found that ion transport-related genes form the largest functional category under selection during salinity change [16,22,24,26]. We found that the same loci (and alleles) are targets of natural selection across salinity gradients in wild populations on different continents and in replicate selection lines in the laboratory [16,26]. In addition, these same ion transport-related genes show coordinated evolutionary shifts in gene expression between saline and freshwater populations [22]. Most notably, parallel selection acting on the same alleles might be driven by positive synergistic epistasis among beneficial alleles and selection on standing genetic variation in the native range populations [16,26,27]. These results suggest that in response to salinity change, a set of cooperating ion transporters undergoes selection and evolves together as a unit.

Given the extraordinary evolutionary capacity of *E*. *affinis* complex populations during invasions, their particular genome architecture might be contributing to this rapid evolutionary response. For instance, specific gene family expansions could enhance the genomic substrate available for natural selection [28]. Additionally, the distribution of critical genes on chromosomes could affect patterns of linkage, which would impact the inheritance of coadapted alleles and their coordinated expression [29]. Furthermore, genome-wide epigenetic signatures, such as the extent and localization of DNA methylation, could impact patterns of gene expression [30]. Despite their potential importance, the role of genome architecture in affecting responses to selection has remained understudied and poorly understood.

Thus, our goal was to generate a high-quality genome sequence for the calanoid copepod *E*. *carolleeae* (Atlantic clade of the *E*. *affinis* species complex) [9,18,31] and begin exploring the genome architecture that might underlie its exceptionally invasive and adaptive capacities. This clade, in particular, is the most invasive within the species complex, with populations currently displacing native Europe clade populations in multiple locations [10–12], and it has the greatest number of freshwater colonizing populations [9]. We assembled a chromosome-level reference genome for this copepod, based on high-coverage Pacific Biosciences (PacBio) long-read, Illumina short-read, and high-throughput chromosome conformation capture (Hi-C) sequencing. To reduce the high level of heterozygosity present in the wild population [16], we generated an inbred line through 30 generations of full-sib mating of a saline population from the St. Lawrence Estuary salt marsh (Baie de L’Isle-Verte). As a result, this newly assembled genome is far more contiguous than our prior assembly, which was based only on Illumina sequencing of the same inbred line [32].

We find in this study that the genome sequence of *E*. *carolleeae* displays an unusual genome architecture underlying the genetic targets of natural selection during salinity change, particularly at the ion transport-related genes. In addition, this genome provides a valuable resource, as the first chromosome-level genome assembly for a calanoid copepod. Moreover, this genome assembly represents one of the most contiguous copepod genomes to date and is among the highest quality marine invertebrate genomes. Only four chromosome-level genome assemblies are available for copepods in the National Center for Biotechnology Information (NCBI) Genome database, namely for two parasitic copepods (order Siphonostomatoida) and two species of the intertidal copepod *Tigriopus* (order Harpacticoida), whereas none are available for the copepod orders Calanoida and Cyclopoida. This deficit of genomic resources for copepods has been quite striking, given their enormous ecological roles as grazers of the sea and their contribution of ∼ 70% of the total zooplankton biomass [33]. The *E*. *affinis* complex in particular has long served as a critically important model system for evolutionary, physiological, and ecological studies, with over 1000 studies published on this copepod system (as of August, 2023, Google Scholar). Moreover, dissecting the peculiar genome architecture of this copepod provides novel insights into its incredible capacity to invade novel environments.

## Results

### High-quality chromosome-level genome assembly of *E*. *carolleeae*

**Figure 1:**
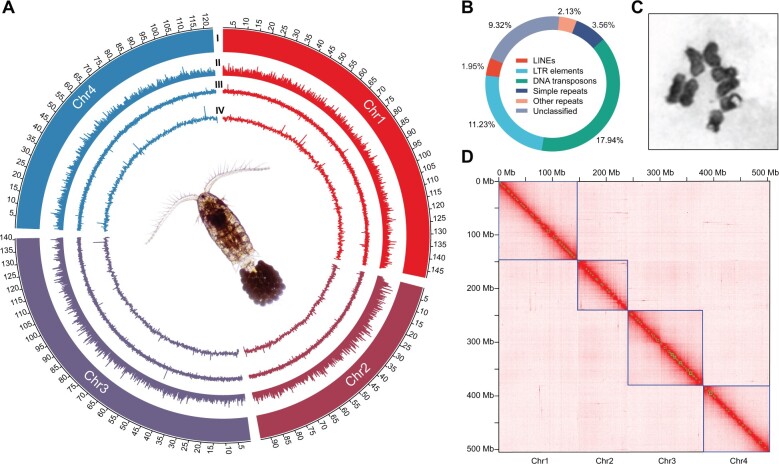
Chromosome-level genome assembly of the copepod *Eurytemora carolleeae* (Atlantic clade of the *Eurytemora affinis* species complex) **A**. Circos plot showing the genomic landscape, including: (I) four chromosomes on the Mb scale; (II) density of protein-coding genes; (III) distribution of GC content (mean GC = 33.0%); and (IV) distribution of repetitive sequences. All distributions were calculated with 100-kb non-overlapping sliding windows, except for the distribution of GC content, which was calculated with 10-kb sliding windows. **B**. Circular diagram showing proportion of different categories of repetitive sequences identified in the copepod genome, with the numbers on the diagram indicating their percentage of occupied length in the genome assembly. Repetitive sequences comprise 46.1% of this copepod genome. **C**. Well-isolated cell showing the karyotype of *E*. *carolleeae* (2*n* = 8) at metaphase. **D**. Hi-C contact map of the genome generated by Juicebox. Chr, chromosome; LINE, long interspersed nuclear element; LTR, long terminal repeat; Hi-C, high-throughput chromosome conformation capture.

The genome assembly that we generated for *E*. *carolleeae* (Atlantic clade of the *E*. *affinis* complex) [18] had a much higher degree of completeness and contiguity than other available copepod genomes (Table S1). Our genome assembly integrated sequence data for an inbred line (30 generations of full-sib mating), using PacBio continuous long-read (CLR) sequencing (∼ 60.6× coverage), PacBio high-fidelity circular consensus sequencing (HiFi CCS) (∼ 14.2× coverage), and Illumina short-read sequencing (∼ 73.4× coverage). These sequence data generated a 536-Mb assembly of 325 contigs, with a contig N50 of 4.2 Mb. This assembly was consistent with the estimated genome size of 509–540 Mb based on *k*-mer analyses (Figure S1). This assembly was further scaffolded based on Hi-C data of ∼ 85.6× coverage and filtered to generate a 529.3-Mb final assembly, with a scaffold N50 of 140.6 Mb. 95.6% of the assembly was anchored onto four pseudo-chromosomes (**Figure 1**A). This genome was highly AT-rich, with a mean GC content of 33.0% (Figure 1A, circle III). This GC content was comparable to those of other calanoid copepods, but lower than those of harpacticoid copepods (Table S1). The GC content of this genome was also lower than that of *Drosophila melanogaster* (42.0%) and lower than 128 out of 154 published genome assemblies of marine invertebrates in a recent survey [34]. The Benchmark of Universal Single-Copy Orthologs (BUSCO) analyses indicated that 93.1% (90.2% single-copy and 2.9% duplicated) of complete BUSCOs (1013 in arthropoda_odb10 dataset) were captured in this genome.

This new genome is vastly superior to our prior assembly based on only Illumina sequencing of the same inbred line [32]. In this new genome, the contig N50 was greatly improved (from 67.7 kb to 4.2 Mb) and the sequences were successfully scaffolded onto chromosomes. The contig N50 length that we obtained here was greater than 33 out of 35 available genome assemblies for copepod species in the NCBI Genome database. The two copepod assemblies with greater contig N50 length than ours were based on Oxford Nanopore Technologies (ONT) sequencing, and their samples were taken from wild outbred populations, rather than inbred lines. The contig N50 of our genome was also longer than 151 out of 154 published genome assemblies of marine invertebrates in a recent survey [34]. Thus, this genome is one of the most contiguous copepod genomes to date and also one of the highest quality marine invertebrate genomes.

### *E*. *carolleeae* karyotype and genome size in the context of copepod evolutionary history

**Figure 2:**
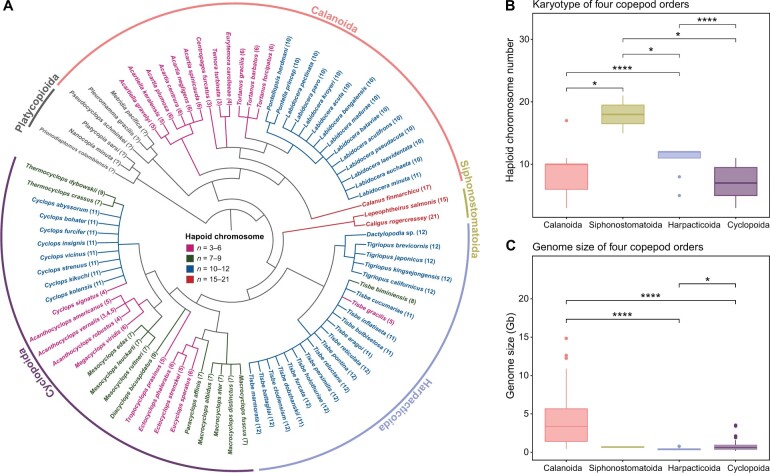
Chromosome number and genome size evolution in the class Copepoda **A**. Synthetic phylogenic topology of copepod species from five copepod orders. This topology was obtained from the synthesis tree of copepods, which integrated 31 published phylogenies, reflecting the collective understanding of copepod relationships to date [78]. Chromosome numbers are shown within parentheses after the species names. The colors of species names indicate the ranges of chromosome numbers. Clades that occupy basal phylogenetic positions, but possess unknown karyotype, are shown in gray in the phylogeny. **B**. Median chromosome number of four copepod orders (see Table S2 for details). Chromosome number differs significantly among the four orders (Kruskal–Wallis test, H = 35.52, DF = 3, *P* = 9.5E−8). **C**. Median genome size of four copepod orders. Median genome sizes for Calanoida, Siphonostomatoida, Harpacticoida, and Cyclopoida are 3179 Mb, 563 Mb, 276 Mb, and 509 Mb, respectively (see Table S3 for details). Genome size differs significantly among the four orders (Kruskal–Wallis test, H = 49.58, DF = 3, *P* = 9.8E−11). Significant differences were determined by Wilcoxon signed rank tests (*, *P* < 0.05; ****, *P* < 1E−4). Nonsignificant *P* values are not shown. DF, degree of freedom.

Our *E*. *carolleeae* genome assembly based on Hi-C data revealed only four haploid chromosomes (2*n* = 8) (Figure 1A–D). Our karyotyping procedure confirmed the presence of four haploid chromosomes in several well isolated cells (Figure 1C, Figure S2). This number is near the lower end for copepods. Chromosome number varies widely among copepod species (2*n* = 6–42) (**Figure 2**A and B; Table S2) and differs significantly among the four copepod orders (Figure 2B) [Kruskal–Wallis test, H = 35.52, degree of freedom (DF) = 3, *P* = 9.5E−8]. While it appears that chromosome number increased during the evolutionary history of the order Calanoida, this pattern is unclear due to the unavailability of karyotype information for the most basal clade within the order Calanoida and the basal clade within the order Platycopioida (Figure 2A, gray clades) in the class Copepoda.

Evolutionary patterns of genomic rearrangements among copepod species are difficult to discern due to lack of synteny between the genome of *E*. *carolleeae* and two other chromosome-level genomes from different copepod orders, namely, the tidepool copepod *Tigriopus californicus* (Harpacticoida) and the salmon louse *Lepeophtheirus salmonis* (Siphonostomatoida) (Figure S3). Only 7 syntenic blocks containing 32 pairs of conserved genes were found between *E*. *carolleeae* and *T*. *californicus* (Figure S3). In contrast, the tidepool copepod and salmon louse genomes showed much greater synteny with each other, sharing 136 syntenic blocks containing 651 pairs of genes, than with *E*. *carolleeae* (Figure S3). Despite this greater synteny, a large number of chromosomal translocations between their genomes were still evident. This lack of synteny among the three copepod genomes indicates that major genomic rearrangements occurred during the course of copepod evolution, with far less conservation relative to vertebrates and some insects, such as butterflies and moths [35,36].

Among copepods, *E*. *carolleeae* has a relatively small genome size. The genome size of *E*. *carolleeae* (1C = 529.3 Mb) is smaller than the average genome size of calanoid copepods (1C = 3993 Mb; based on data from 41 species). This small genome size makes *E. carolleeae* an outlier among calanoid copepods (Figure 2C). The *E. carolleeae* genome is also smaller than the average genome size of 1.85 Gb for 112 copepod species across four orders (as detailed in Table S3). Overall, the range in genome size among copepod species is large (1C = 0.1–14.4 Gb) (Tables S1 and S3), with significant differences among the four copepod orders (Figure 2C) (Kruskal–Wallis test, H = 49.58, DF = 3, *P* = 9.8E−11).

### Comprehensive genome annotation and comparative analysis of *E*. *carolleeae*

A total of 20,262 protein-coding genes were predicted in the *E*. *carolleeae* genome, occupying 261.62 Mb in length of the genome assembly (Table S4). This estimate was based on abundant transcriptome data for the *E*. *affinis* complex, homologous proteins of other arthropods, and *ab initio* prediction. Among these genes, almost all genes (20,259) were functionally assigned based on at least one of eight functional annotation databases (Table S5). This predicted number of annotated protein-coding genes was greater than that of the tidepool copepod *T*. *californicus* (15,500 genes) and the salmon louse *L*. *salmonis* (13,081 genes).

The higher number of genes in our genome was not due to gene fragmentation, as indicated by our mean gene length of 12.91 kb, mean coding sequence length of 1.45 kb, and mean exon number per gene of 10.9 (Table S4). In addition, this high number of genes was not due to counting separate alleles as genes, given that we used an inbred line with heterozygosity of ∼ 0.5% (Figure S1) and the duplicated BUSCO detected in the genome assembly was only 2.9%.

Our estimation of high gene count in the *E*. *carolleeae* genome was neither a consequence of errors in genome assembly or gene prediction nor a consequence of whole genome duplication (WGD). To determine whether genome assembly and gene prediction errors led to the overestimation of gene count, we assessed the read depth across all predicted genes. Genes exhibiting very low to no coverage could potentially contribute to errors in gene prediction. Our analysis revealed substantial read depth across genes, ranging from 80 to 113,714, with an average of 8972 reads for each predicted gene, supporting the accuracy of our gene predictions. Among the predicted genes, we identified 977 genes (∼ 5%) exhibiting exceptionally high read depths that deviated significantly from the central tendency based on the interquartile range (IQR) method. Such high read depths suggest gene duplication and expansion events within the genome. To determine whether the greater gene number was caused by ancient WGD events, we examined the distribution of synonymous substitutions per site (*Ks*) among paralogous genes within the genome (known as *Ks* plot analysis). Based on the *Ks* plot, we found no evidence of ancient WGD in the *E*. *carolleeae* genome (Figure S4). Interestingly, the largest proportions of gene duplication events occurred quite recently (*Ks* = 0–0.04) (Figure S4).

By integrating our *de novo* repetitive sequence database with public repetitive sequence databases of arthropods, we identified 46.1% of the *E*. *carolleeae* assembly as repetitive sequences, which comprised 244.1 Mb in length of the genome assembly (Figure 1A, circle IV). DNA transposons and long terminal repeat (LTR) elements comprised the largest percentages of repetitive sequences in the *E*. *carolleeae* genome (Figure 1B; Table S6). Among the four copepod genomes (*E*. *carolleeae*, *T*. *californicus*, *L*. *salmonis*, and *Caligus rogercresseyi*), a strong positive linear correlation was detected between genome size and proportion of repetitive sequences (Pearson correlation coefficient *r* = 0.914) (Figure S5A and B). In the four copepod genomes, the Gypsy/DIRS1 superfamily constituted the largest proportion of LTR elements, while the hobo-Activator and Tc1-IS630-Pogo superfamilies were predominant among the DNA transposons (Figure S5C and D). Compared to the other three copepod species, the *E*. *carolleeae* genome possessed the highest proportion of simple sequence repeats (SSRs) and the lowest proportion of long interspersed nuclear elements (LINEs) (Figure S5B).

We identified and annotated 2426 non-coding RNA (ncRNA) sequences in the genome, among which 1574 transfer RNA (tRNA) sequences formed the largest category (Table S7). This number of ncRNA sequences was within the range of 386–4559 in other copepod genomes in the NCBI Genome database.

### Dynamics of gene family evolution in *E*. *carolleeae* and across the Arthropoda

**Figure 3:**
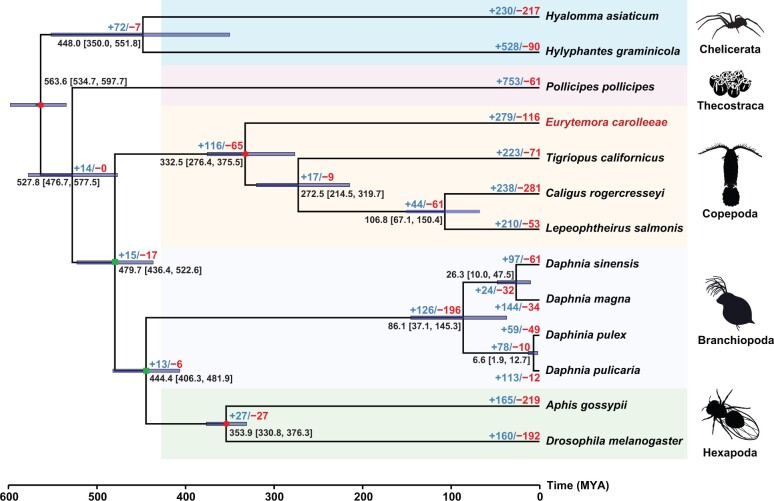
Gene family expansions and contractions during the evolutionary history of the Arthropoda with a focus on the Copepoda Phylogenetic reconstruction of 13 high-quality arthropod genomes was performed using RAxML based on concatenated single-copy orthologous genes. All nodes show bootstrap values of 100%, except for two nodes with green rectangles, which have values of 66% (left node) and 60% (right node). The three red dots represent time calibrated nodes, with confidence time intervals retrieved from the TimeTree database and applied in MCMCTree. Mean estimated divergence time is shown at each node with numbers in brackets indicating the interval containing the 95% highest posterior densities. The numbers of expanded gene families (in blue) and contracted gene families (in red) are shown on the branch tips and next to each node. MYA, millions of years ago.

To determine patterns of gene family gains and losses in the *E*. *carolleeae* genome and across the Arthropoda, we conducted a comparative genomic analysis using shared ortholog groups (gene families) across 12 additional arthropod species. In this comparative analysis, we included only high-quality genomes from different arthropod subphyla, assembled with long-read sequencing data to the chromosome level. A phylogeny was reconstructed using a matrix of 101 concatenated single-copy orthologous genes (Table S8). This phylogeny supported the topology of ((((Hexapoda + Branchipoda) + Copepoda) + Thecostraca) + Chelicerata), although the relationships between Hexapoda, Branchiopoda, and Copepoda were not highly supported (**Figure 3**, green dots at nodes). Overall, we found substantial numbers of conserved orthologous genes (4042) shared among *E*. *carolleeae* and three other pancrustacean species (Figure S6).

**Figure 4:**
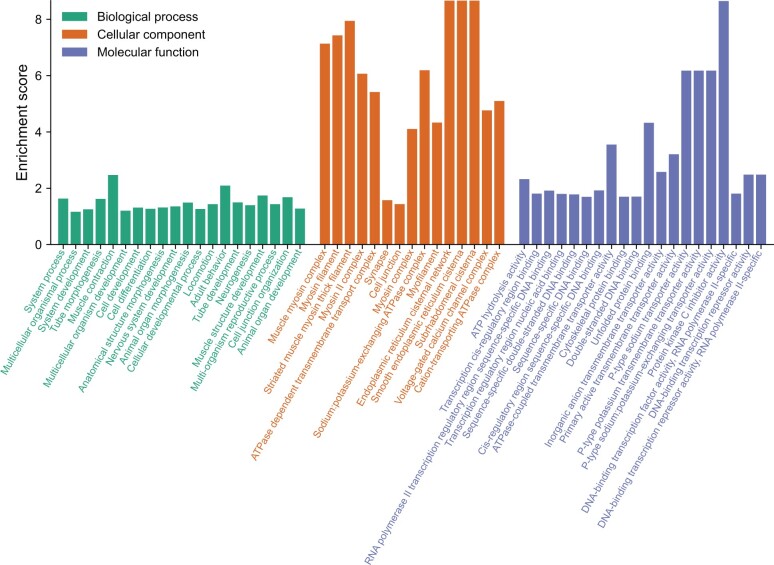
Significantly enriched GO terms in the expanded gene families in the *E*. *carolleeae* genome The GO terms were sorted by *P* value (with higher *P* values toward the right in each category). The complete list of enriched GO terms is shown in Table S11. Only the top 20 GO terms of the biological process and molecular function categories, and top 15 GO terms of cellular component category are shown here. GO, Gene Ontology.

Our analysis of gene family expansions and contractions revealed a significant enrichment of ion transport-related genes in the *E*. *carolleeae* genome (**Figure 4**, Figure S7; Tables S9–S12). Relative to other arthropod genomes, this copepod genome displayed the expansion of 279 ortholog groups (also known as gene families) containing 1161 genes (Table S9), and the contraction of 116 gene families comprising 224 genes (Figure 3; Table S10).

Through gene function enrichment analysis, we found that 29.2% (61 out of 209) of the significantly enriched Gene Ontology (GO) terms in the molecular function (MF) category was related to ion transport activity. Among them, 63.9% (39 out of 61) were related specifically to inorganic ion (cation and anion) transport activity (Figure 4, Figure S7; Tables S11 and S12). In the cellular component (CC) category, 7.6% (11 out of 144) of the significantly enriched GO terms were related to ion transport activity, whereas in the biological process (BP) category, 5.6% (98 out of 1734) of the significantly enriched GO terms were related to ion transport and regulation of ion transporter activity. The most significantly enriched GO terms in the MF category included “ATPase-coupled transmembrane transporter activity” (GO:0042626), “inorganic anion transmembrane transporter activity” (GO:0015103), “primary active transmembrane transporter activity” (GO:0015399), “P-type sodium transporter activity” (GO:0008554), “P-type potassium transmembrane transporter activity” (GO:0008556), and “P-type sodium:potassium-exchanging transporter activity” (GO:0005391) (Figure 4). Similarly, in the CC category, the most significantly enriched GO terms included “ATPase dependent transmembrane transport complex” (GO:0098533), “sodium:potassium-exchanging ATPase complex” (GO:0005890), and “cation-transporting ATPase complex” (GO:0090533) (Figure 4). In the BP category, significant GO terms included “regulation of sodium ion transmembrane transporter activity” (GO:2000649, GO:1902305), “regulation of sodium ion export across plasma membrane” (GO:1903276), and development-related categories, such as “cell development” (GO:0048468) and “cellular developmental process” (GO:0048869) (Table S11).

The significantly expanded gene families in the *E*. *carolleeae* genome (Figure 3; Table S9) included ion transport-related gene families that we found in previous studies to be repeatedly under natural selection during salinity change in *E*. *affinis* complex populations [16,22,24,26,37]. These gene families included Na^+^/K^+^-ATPase α subunit (*NKA-α*), Na^+^/K^+^-ATPase β subunit (*NKA-β*), and solute carrier family 4 (*SLC4*) of the bicarbonate (${\text{HCO}}_{3}^{-}$) transporters [including anion exchanger (*AE*), Na^+^,${\text{HCO}}_{3}^{-}$ cotransporter (*NBC*), and Na^+^-driven Cl^−^/${\text{HCO}}_{3}^{-}$ exchanger (*NDCBE*)].

Of the ion transport-related gene families under selection during salinity change [16,22,24,26,37], many had greater numbers of gene paralogs than other gene families in the *E*. *carolleeae* genome. For instance, the ion transport-related gene families under selection [16,22,24,26,37] had a mean paralog number of 7.6 (Table S13), which was higher than the mean paralog number of 4.2 for all expanded gene families in the *E*. *carolleeae* genome. Substantial read depth, ranging from 917 to 34,710 with an average of 8834, was observed for these ion transport-related genes, affirming the reliability of their gene predictions (Table S13).

Interestingly, many of the ion transporter gene families showed signatures of very recent gene duplications. In particular, the ion transporter gene families *NKA-α*, *NKA-β*, ammonia transporter (*AMT*), and vacuolar-type H^+^-ATPase (*VHA*) subunit a were contained within the most recent category of gene duplications (*Ks* = 0.00002–0.09 substitutions per synonymous site) based on a *Ks* distribution analysis (Figure S4; Table S13).

### Genome-wide CpG_o/e_ depletion in gene bodies as signatures of gene body methylation

Next, we analyzed the genome-wide signatures of CpG depletion to gain insights into historical patterns of methylation throughout the *E*. *carolleeae* genome [30,38,39]. This approach allowed us to uncover long-term and stable methylation signatures that reflect evolutionary responses to past environments [38,40,41]. That is, we could identify methylation patterns that have been consistently important for *E*. *carolleeae* populations to thrive in changing environments, including changing salinity. Thus, unlike current methylation profiles, which can be highly variable and context-specific, exploring CpG depletion patterns can provide a fundamental understanding of how past methylation patterns have been established and maintained over longer evolutionary timescales.

**Figure 5:**
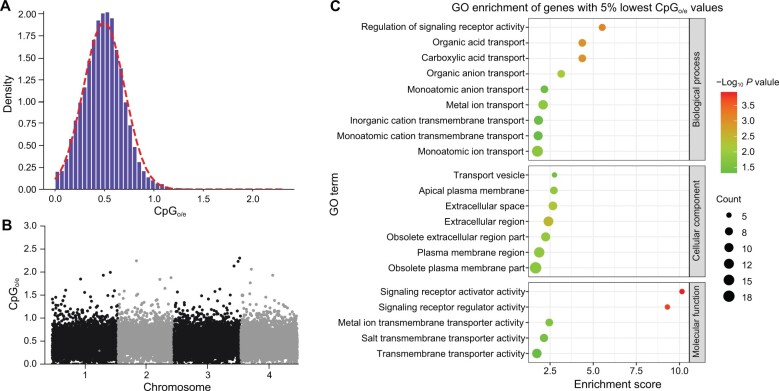
Patterns of genome-wide CpG_o/e_ values of gene bodies corresponding to signatures of past gene body methylation in the *E*. *carolleeae* genome **A**. Distribution of genome-wide CpG_o/e_ values of protein-coding genes in the *E*. *carolleeae* genome, showing a unimodal distribution with a low mean value of 0.5. **B**. Distribution of CpG_o/e_ values across the genome, showing gene positions on each chromosome. **C**. GO enrichment of 1013 genes with 5% lowest CpG_o/e_ values. Significance levels of GO enrichment are shown by the color of circles and numbers of enriched genes are indicated by the size of circles. The ion transport-related genes tend to have the lowest CpG_o/e_ values, suggesting extremely high levels of historical gene body methylation [49]. CpG_o/e_, the ratio between the observed and expected incidences of CpG sites (where a cytosine is followed by a guanine).

The *E*. *carolleeae* genome exhibited striking genome-wide signatures of CpG depletion in gene bodies, indicating high levels of historical gene body methylation across the genome. Notably, the most CpG-deficient genes tended to be ion transport-related genes (**Figure 5**). DNA methylation of gene bodies is typically associated with increased expression levels and/or greater regulation of gene expression, although this relationship might not be always positive [42–47]. We determined the genome-wide distribution of CpG sites, to determine genome-wide signatures of historical DNA methylation of the predicted protein-coding genes. We calculated CpG_o/e_ values, which are the ratios between the observed and expected incidences of CpG sites [where a cytosine (C) is followed by a guanine (G)]. Typically, genes with lower CpG_o/e_ values (lower numbers of observed CpG sites than expected) likely have undergone higher levels of DNA methylation in the past (see Discussion) [38,40,41].

The CpG_o/e_ values across all genes displayed a unimodal distribution, with a very low mean CpG_o/e_ value of 0.5 in the *E*. *carolleeae* genome (Figure 5A). This unimodal distribution and low mean CpG_o/e_ value represent an extreme case of CpG depletion, indicating genome-wide signatures of high levels of historical gene body methylation [39]. Most of the genes (19,960 out of 20,262) had CpG_o/e_ values lower than 1 (Figure 5A), and the distribution of CpG_o/e_ values was not biased by the positions of genes on different chromosomes (Figure 5B). The mean CpG_o/e_ value of the *E. carolleeae* genome was much lower than that of the *D*. *melanogaster* genome (mean CpG_o/e_ value around 1), which also displayed a unimodal distribution [30]. Moreover, the unimodal distribution observed in the *E. carolleeae* genome differed from the bimodal distributions found in the genomes of many molluscs [39] and insects [30,38]. Based on our genome annotation, the *E*. *carolleeae* genome does contain genes that encode enzymes that perform DNA methylation, namely, DNA methyltransferases DNMT1 and DNMT2, but not DNMT3. In contrast, these genes are lacking in the genomes of *D*. *melanogaster* and some other model organisms, such as yeast *Saccharomyces cerevisiae* and the nematode worm *Caenorhabditis elegans* [30,48].

GO enrichment analysis to determine functions of genes with the 5% lowest and 5% highest CpG_o/e_ values (1013 genes) revealed very different sets of gene functions in the two groups. Notably, genes with the 5% lowest CpG_o/e_ values were significantly enriched with GO terms related to ion transmembrane transport functions (Figure 5C; Table S14). Specifically, 66.7% (6 out of 9) GO terms in the BP category and 60% (3 out of 5) GO terms in the MF category were related to ion transport (Figure 5C). These GO terms included “monoatomic anion transport” (GO:0006820), “monoatomic ion transport” (GO:0006811), “inorganic cation transmembrane transport” (GO:0098662), “metal ion transmembrane transporter activity” (GO:0046873), and “salt transmembrane transporter activity” (GO:1901702). These low CpG_o/e_ values for ion transport-related genes suggest that these genes had extremely high levels of gene body methylation in the past [49].

Specifically, the mean CpG_o/e_ value for 490 ion transport-related genes was 0.47, similar to the low genome-wide mean value of 0.5 in the *E*. *carolleeae* genome (Figure 5A). Likewise, for 80 key candidates among the 490 ion transport-related genes that were identified as targets of natural selection during salinity transitions in *E*. *affinis* complex populations [16,22,24,26,37], the CpG_o/e_ values ranged between 0.09 and 0.80 [mean = 0.45, standard deviation (SD) = 0.17] (Table S13). Of these 80 key ion transport-related genes, 66.3% (53 out of 80) possessed CpG_o/e_ values lower than the mean CpG_o/e_ value of 0.5 in the *E*. *carolleeae* genome (Figure 5A), indicating even higher levels of historical gene body methylation for many of the ion transport-related genes that showed signatures of selection in our previous studies [16,22,24,26,37].

In contrast, genes with the highest CpG_o/e_ values were enriched with conserved cellular functions, such as “nucleic acid binding” (GO:0003676), “RNA processing” (GO:0006396), and “RNA metabolic process” (GO:0016070) (Table S15). These GO terms represent housekeeping genes, with our results suggesting relatively low levels of past methylation. In contrast, these genes were identified as hypermethylated in previous studies on insects [30,38].

### Localization of ion transport-related genes on the four chromosomes

**Figure 6:**
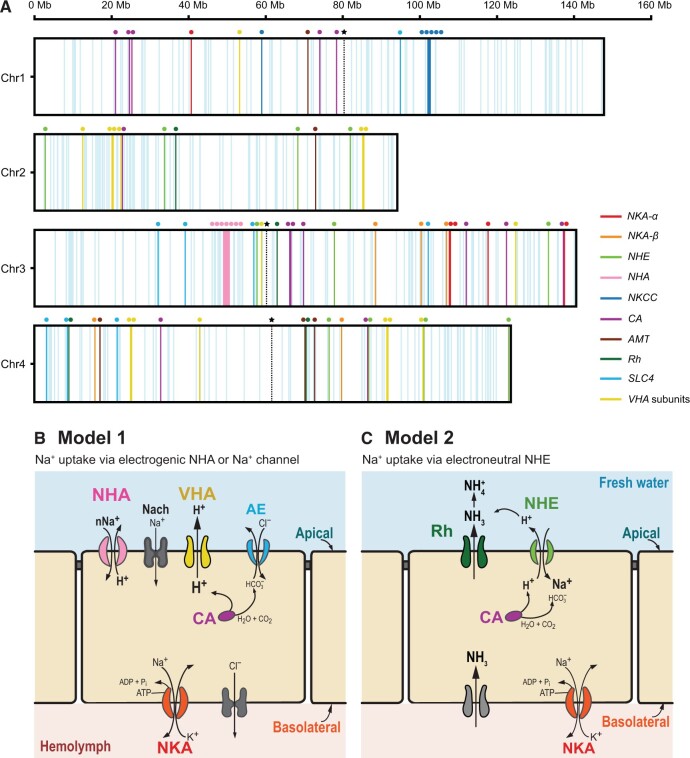
Localization of ion transport-related genes on *E*. *carolleeae* chromosomes and hypothetical models of ion uptake from fresh water **A**. Ion transport-related genes were mapped onto the four *E*. *carolleeae* chromosomes. All vertical lines represent the 490 genes with ion (cation and anion) transporting function based on genome annotation. The vertical lines marked with colored dots represent the 80 key ion transport-related genes, which showed evolutionary shifts in gene expression and/or signatures of selection in prior studies, and are likely involved in the hypothetical models of ion uptake (in B and C, adapted from [50]). The vertical dashed lines marked with stars indicate the positions of centromeres based on the Hi-C contact map (Figure 1D, Figure S12). **B**. Hypothetical model 1 of ion uptake from freshwater environments. VHA generates an electrochemical gradient by pumping out protons, to facilitate uptake of Na^+^ through an electrogenic Na^+^ transporter (likely NHA). CA produces protons for VHA. **C**. Hypothetical model 2 of ion uptake from freshwater environments. An ammonia transporter Rh protein exports NH_3_ out of the cell and then this NH_3_ reacts with H^+^ to form ${\text{NH}}_{4}^{+}$. The resulting deficit of extracellular H^+^ concentrations causes NHE to export H^+^ in exchange for the import of Na^+^. CA produces protons for NHE. These models are not comprehensive for all tissues or taxa and are not mutually exclusive. NKA-α, Na^+^/K^+^-ATPase α subunit; NKA-β, Na^+^/K^+^-ATPase β subunit; NHE, Na^+^/H^+^ exchanger; NHA, Na^+^/H^+^ antiporter; NKCC, Na^+^,K^+^,Cl^−^ cotransporter; CA, carbonic anhydrase; AMT, ammonia transporter; Rh, Rh protein; SLC4, solute carrier family 4 of bicarbonate transporters; AE, anion exchanger; NBC, Na^+^,${\text{HCO}}_{3}^{-}$ cotransporter; NDCBE, Na^+^-driven Cl^−^/${\text{HCO}}_{3}^{-}$ exchanger; VHA subunits, subunits of vacuolar-type H^+^ ATPase; Nach, sodium channel.

Given that ion transport-related genes were the most enriched GO category in the *E*. *carolleeae* genome, we manually annotated and localized the 490 ion transport-related genes on the four chromosomes (**Figure 6**A; Table S13).

We focused heavily on the 80 key ion transport-related genes that were identified as targets of natural selection during salinity transitions in *E*. *affinis* complex populations [16,22,24,26,37] and likely involved in hypothesized models of ion uptake in freshwater habitats (Figure 6B and C) [50]. For instance, these 80 ion transport-related genes that we mapped onto the chromosomes included the gene families *NKA-α*, *NKA-β*, Na^+^/H^+^ antiporter (*NHA*), Na^+^/H^+^ exchanger (*NHE*), Na^+^,K^+^,Cl^−^ cotransporter (*NKCC*), carbonic anhydrase (*CA*), *AMT*, Rh protein (*Rh*), subunits of *VHA*, and *SLC4* of bicarbonate (${\text{HCO}}_{3}^{-}$) transporters (including *AE*, *NBC*, and *NDCBE*) [24]. We found unequal numbers of these 80 ion transport-related genes on each chromosome, with 15, 13, 30, and 22 key genes found on Chr1 to Chr4, respectively (Figure 6A). Interestingly, the highest density of these key ion transport-related genes was localized on the second longest chromosome, Chr3, which contained two-fold the number of key genes than the longest Chr1.

We found that the distribution of ion transport-related genes on the chromosomes deviated significantly from a uniform distribution and tended to be more clustered than expected, for all 490 genes with putative ion transporting function based on genome annotation (Kolmogorov–Smirnov test, D = 0.71, *P* = 1.1E−25) (Figure 6A, Figure S8A), as well as for the 80 key ion transport-related genes found under selection (Kolmogorov–Smirnov test, D = 0.76, *P* = 3.2E−30) (Figure 6A, Figure S8B). In addition, the distribution of ion transport-related genes differed significantly from those of functionally conserved housekeeping genes (Tables S15 and S16), and showed a higher frequency of closely spaced genes (Figure S9), for all 490 genes found with ion transporting function (Chi-square goodness of fit test, χ^2^ = 73.0, DF = 15, *P* = 1.3E−9) (Figure S9A), as well as for the 80 key ion transport-related genes (Chi-square goodness of fit test, χ^2^ = 37.9, DF = 5, *P* = 3.9E−7) (Figure S9B). Notably, we found a high density of key ion transport-related genes clustered around the centromere of Chr3 (Figure 6A, Figures S10 and S11). Centromeres were identified based on the Hi-C contact map (Figure 1D, Figure S12).

## Discussion

Copepods form the largest animal biomass on the planet and contribute to the majority of total zooplankton biomass in aquatic habitats [1,33]. However, despite their critical roles for ecosystem functioning and maintenance of fisheries on the planet, high-quality genomic resources are relatively lacking. Among copepods, populations of the *E*. *affinis* species complex are notable for their extremely high abundance and critical importance as grazers in coastal ecosystems [5–8]. Intriguingly, some populations have greatly expanded their ranges in recent years into novel habitats, particularly into novel salinities [9,15,24].

Our results on the genome of the copepod *E*. *carolleeae* (Atlantic clade of the *E*. *affinis* complex) reveal a genome architecture that likely enables its populations to be particularly responsive to changes in habitat salinity. The particular genomic features found here that likely contribute to responses to salinity include the extensive and recent expansions of ion transport-related gene families, the extremely high levels of historical methylation of ion transport-related gene bodies, and the physical clustering of ion transport-related genes. These genomic features contain many of the ion transport-related genes that showed signatures of selection during salinity change in our previous studies [16,22,24,26,37]. Such genomic features potentially play integral roles in the extraordinary ability of populations of the *E*. *affinis* species complex to invade biogeographic boundaries into novel salinities [9,51].

### Distinctive features of the first calanoid copepod reference genome

This study presents the first chromosome-level reference genome of a calanoid copepod, specifically *E*. *carolleeae* from the Atlantic clade of the *E*. *affinis* species complex. Characterized by high completeness and contiguity, this genome stands as one of the highest quality marine invertebrate genomes, setting a new standard for copepod genomic research [9,18,31,34]. Such high-resolution genomic data provide invaluable resources for future studies of this ecologically critical group.

The genome of *E*. *carolleeae* is notably compact, with a size of 529.3 Mb and a diploid chromosome count of 8, which are both relatively low compared to other copepod genomes (Tables S2 and S3). This relatively small genome size of *E*. *carolleeae* might be a result of its large effective population size in nature [52]. The effective population size of *E*. *carolleeae* in the St. Lawrence Estuary is approximately 1 × 10^6^ based on our previous estimates of Watterson’s theta (0.0131) [16], assuming a mutation rate of 3.46 × 10^−9^ based on *D*. *melanogaster* [53].

The *E*. *carolleeae* genome size in this present study aligns with our previous estimates [32] for the same inbred line (based on 30 generations of inbreeding of a population from Baie de L’Isle-Verte, Quebec, Canada), confirming the stability and reproducibility of our genome assembly. Our earlier draft genome sequence assembled from Illumina sequences [32], based on DNA exclusively from egg sacs (embryonic tissue) from the same inbred line, yielded a similar estimated genome size of ∼ 510 Mb (Figure S1). Our estimates based on DNA cytophotometry of embryonic cells from the same population yielded a 2C genome size of 0.6–0.7 pg DNA/cell or 1C = 318 Mb [54].

Chromosome number varies substantially among copepod species, indicating an evolutionary history marked by chromosomal fusions and fissions and associated genomic rearrangements (Figure 2B; Tables S2 and S3). The variation in chromosome number in copepods exceeds the levels found in vertebrates and insects [35,36]. Such variation likely contributes to the low levels of synteny among copepod genomes (Figure S3). In addition, many of the gene family expansions in the *E*. *carolleeae* genome might contribute to the disruption of conserved synteny and account for the lower synteny between *E*. *carolleeae* and other copepod genomes (Figure S3). For instance, the tidepool copepod *T*. *californicus* genome lacks the extensive ion transporter gene family expansions observed in *E*. *carolleeae* and contains only one paralog each of the *NHA*, *NKA-α*, *NKA-β*, and *NKCC* gene families.

Moreover, genome size across different copepod species and orders varies considerably (Figure 2C; Table S3), likely influenced by the amount of repetitive sequences (Figure S5). This variation in genome size is particularly pronounced in the copepod order Cyclopoida (Table S3). The relatively large genome sizes (> 1 Gb) of some cyclopoid species reflect only the germline genome and not the somatic genome [55–57]. Some copepods undergo chromatin diminution, which is the programmed deletion of chromatin from embryonic presomatic cells during development, resulting in a 5–75-fold reduction in somatic genome size [55,58]. However, this chromatin diminution is not observed in *E*. *carolleeae* [54].

### Massive expansions of ion transport-related gene families in the *E*. *carolleeae* genome

Our comparative genomic analysis involving 13 high-quality arthropod genomes revealed notable expansions in gene families related to ion transport within the *E*. *carolleeae* genome. These expansions, which make up 29.2% of GO MF terms (Figures 3 and 4; Tables S9 and S11), suggest an extensive osmoregulatory capacity that likely enhances the copepod’s ability to adapt to changing salinities.

Ion transport-related genes have been found repeatedly to constitute the largest functional (GO) categories under selection during salinity change in our previous evolutionary and physiological studies of *E*. *affinis* complex populations (vertical lines with colored dots in Figure 6A) [15,16,24,26,37]. We found that these ion transport-related genes were under selection across multiple independent saline to freshwater invasions in North America [16] and across salinity clines in wild populations in the Baltic Sea [37]. We also found significant signatures of selection at these genes in replicate experimental lines during ten generations of laboratory selection for low salinity tolerance [26]. Additionally, our physiological studies have localized the expression of some of these ion transporter proteins in the maxillary glands, swimming legs, and digestive tract of *E*. *affinis complex* copepods [23,59,60]. We found that the expression and activity of these ion transporters have evolved between saline and freshwater populations and shown acclimatory shifts across salinities [21–23].

Intriguingly, the ion transport-related gene families with signatures of selection exhibited unusually high numbers of paralogs (mean = 7.6) compared to all expanded gene families throughout the *E*. *carolleeae* genome (mean = 4.2). Moreover, low divergence times among the gene paralogs (*Ks* = 0.00002–0.09) (Figure S4) indicate that many of the ion transporter gene families (*i.e.*, *NKA-α*, *NKA-β*, *AMT*, and *VHA* subunit a) show evidence of recent genome duplications. This recent diversification within roughly 10^4^–10^8^ generations points to relatively recent evolutionary responses to changing environments, possibly facilitating niche adaptation across salinity gradients.

These massively expanded ion transport-related gene families likely provide the potential for functional differentiation among the ion transport-related paralogs. Given the negative genetic correlations between saline and freshwater tolerance in *E*. *affinis* complex populations [14,61], it is quite possible that different ion transport-related paralogs are functioning optimally at different salinities. Such functional differentiation could provide greater versatility in acclimatory responses and valuable genetic substrate for natural selection in the face of salinity change. In fact, a previous study has found that different ion transport-related paralogs (*e.g.*, *NHA*, *NKA*, *CA*, and *NKCC*) show considerable variation in acclimatory and evolutionary shifts in gene expression in response to salinity change [22]. In addition, ion transport-related paralogs vary in their signatures of selection across salinity gradients in wild populations and during salinity decline in laboratory selection lines [16,24,26,37]. More studies are required to determine functional differences among the ion transport-related paralogs and how acclimatory and selection responses differ among them.

### Genome-wide patterns of historical methylation of ion transport-related genes in *E*. *carolleeae*

Our analysis of CpG depletion across the *E*. *carolleeae* genome reveals extensive signatures of historical methylation, particularly in ion transport-related gene bodies. In the *E*. *carolleeae* genome, the extremely low genome-wide CpG_o/e_ values and unimodal distribution (Figure 5A and B) suggest high levels of past genome-wide gene body methylation of most genes in the genome (with the highest levels of methylation at the ion transport-related genes). This pattern might reflect long-term evolutionary responses to varying salinity conditions. Unlike the detection of contemporary patterns of methylation marks, signatures of historical methylation have accumulated over extended evolutionary time scales and could offer insights into long-term adaptive responses to environmental fluctuations.

The genome-wide depletion of CpG sites, indicated by a very low mean CpG_o/e_ value of 0.5, suggests pervasive high levels of historical gene body methylation (Figure 5). Remarkably, 98.5% of genes exhibited this depletion pattern, with CpG_o/e_ values below 1. Genes with lower CpG_o/e_ values (lower numbers of observed CpG sites than expected) indicate that they likely have undergone higher levels of DNA methylation in the past. Most DNA methylation events occur at CpG sites and result in the production of 5-methylcytosine (5mC). Subsequently, spontaneous deamination of 5mC leads to C to T conversion [40,49]. Thus, high levels of DNA methylation will eventually cause the depletion of CpG sites associated with genes [38,40,41].

The genome-wide CpG depletion observed here is rare in invertebrate species, but more common in vertebrates [30,39,41,48,62]. The mean CpG_o/e_ value of 0.5 in the *E*. *carolleeae* genome was lower than those of 152 out of 154 arthropod species surveyed [62]. Based on this survey, the mean CpG_o/e_ value for *E*. *carolleeae* was comparable to the lowest CpG_o/e_ value of 0.47 for two species, the fiddler crab *Celuca pugilator* and the remipede crustacean *Xibalbanus tulumensis* [62]. The CpG depletion of the *E*. *carolleeae* genome likely contributes to its low GC content (33.0%).

In addition, the CpG_o/e_ values across all genes in the *E*. *carolleeae* genome displayed a characteristic unimodal distribution. Such a unimodal distribution of CpG_o/e_ values is common in vertebrates, but extremely rare in invertebrates, which tend to have a mosaic pattern of both low and high CpG_o/e_ genes [30,39,41,42,48,62]. This unimodal distribution of low CpG_o/e_ values in the *E*. *carolleeae* genome reflects the unusual pattern of mostly low CpG_o/e_ genes, reflecting high levels of methylation of most genes.

Most notably, many of the ion transport-related genes in the *E*. *carolleeae* genome exhibited the lowest CpG_o/e_ values (Figure 5C; Tables S13 and S14), indicating complete or nearly complete depletion of CpG sites. This result suggests that the ion transport-related genes have experienced extremely high levels of historical gene body methylation. This pattern might be consistent with the critical roles of ion transport-related genes and the need for controlled transcriptional regulation during the evolutionary history of environmental fluctuations of this species complex and perhaps of the genus *Eurytemora* [15,16,24,27,63–67].

Multiple studies have found that DNA methylation of gene bodies is positively correlated with increased levels of gene expression, in contrast to the suppression of gene expression by DNA methylation of gene promoter sequences [42–47]. Gene body methylation has been proposed to facilitate responses to environmental change and assist in acclimation by modulating gene expression [65,68]. Gene body methylation has also been suggested to maintain the transcriptional robustness by preventing aberrant transcription or regulating splicing efficiency, contributing to long-term stress adaptation [66,67]. This preponderance of low CpG_o/e_ genes, particularly ion transport-related genes, is consistent with genome-wide global responses to changing salinity of past environments over extended evolutionary time.

### Clustering of ion transport-related genes on the four chromosomes of the *E*. *carolleeae* genome

The spatial clustering of key ion transport-related genes on the four chromosomes of the *E*. *carolleeae* genome (Figure 6) suggests a genome architecture that could potentially enhance the co-expression and coordinated function of these critical genes. This clustering could be driven by evolutionary pressures that favor the linkage of genes involved in shared physiological pathways (*e.g.*, Figure 6B), thereby enabling more synchronized responses to environmental stressors. For example, the pronounced clustering of 14 key ion transport-related genes proximate to the centromere of Chr3 (Figure S11) might facilitate coordinated acclimatory and adaptive responses to salinity change. The close physical linkage of beneficial alleles, especially at the centromeres, might be favored by selection to reduce recombination [26,29,69], which would tend to separate linked alleles. Such clustering and linkage would facilitate the inheritance of co-adapted alleles as a unit. Thus, such a genomic feature that maintains the clustering of beneficial alleles might serve as a contributing mechanism that facilitates rapid adaptation.

The significant clustering of key ion transport-related genes in the *E*. *carolleeae* genome might be a byproduct of neutral processes, such as recent expansions of ion transport-related genes (previous section; Figure S4). However, the ion transport-related gene family expansions do not appear to account for the high levels of clustering that we observed. Of the 80 key ion transport-related genes that previously showed signatures of selection [16,22,24,26,37], only 16 are tandem paralogs (Figures 6A and S10).

Genomic rearrangements, particularly chromosomal fusions, likely contributed to the clustering of key ion transport-related genes observed in the *E*. *carolleeae* genome [70]. Notably, our subsequent study, comparing the diverse genome architectures of different clades (sibling species) within the *E*. *affinis* species complex, has revealed that chromosomal fusion events from the ancestral karyotype resulted in the joining of key ion transport-related genes in the *E*. *carolleeae* genome, especially at the centromeres [70]. Future studies should explore the functional consequences and selective benefits of clustering these key ion transport-related genes within the *E*. *carolleeae* genome.

## Conclusion

The genomic characteristics described here might be relatively widespread among successful invaders crossing salinity boundaries. A large portion of the most prolific invasive species in freshwater lakes and reservoirs are immigrants from more saline waters, such as zebra mussels, quagga mussels, and many branchiopod and amphipod crustaceans [51,64]. Moreover, the capacity to endure or evolve in response to salinity change is likely to become increasingly critical, as climate change is inducing drastic salinity changes throughout the globe, with rapid salinity declines in high-latitude coastal regions [71]. The high-quality genome assembly of *E. carolleeae* generated in this study provides an invaluable resource for gaining novel insights into genomic mechanisms that might enable rapid responses to environmental change and rapid invasions into novel habitats [72].

## Materials and methods

### Sampling and laboratory inbreeding of *E*. *carolleeae*

A population from the Atlantic clade of the *E*. *affinis* species complex (*E*. *carolleeae*) was originally collected in Baie de L’Isle-Verte, St. Lawrence Estuary, Quebec, Canada (48°00′14″N, 69°25′31″W) in October, 2008. To reduce heterozygosity of the wild population, inbred lines were generated through 30 generations (2.5 years) of full-sib mating. The inbred lines were continuously reared and maintained in multiple 2-l beakers containing 15 PSU (practical salinity unit, approximate parts per thousand salinity) saline water (0.2 μm pore filtered) made with Instant Ocean (Catalog No. SS15-10, Blacksburg, VA), along with primaxin (20 mg/l) to avoid bacterial infection. The copepods were fed with the marine alga *Rhodomonas salina* three times per week with water changed weekly. The inbred line VA-1 was used for this study.

### Sequencing of the *E*. *carolleeae* genome

For genome sequencing, approximately 3000 adult copepods were initially collected. To minimize contamination of the DNA extraction with copepod gut contents and its microbiome, the copepods were treated with antibiotics (20 mg/l primaxin, 0.5 mg/l voriconazole) and D-amino acids (10 mM D-methionine, 10 mM D-tryptophan, 10 mM D-leucine, and 5 mM D-tyrosine) two weeks prior to DNA extraction with water changed twice per week. The copepods were then treated with five additional antibiotics (20 mg/l rifaximin, 40 mg/l sitafloxacin, 20 mg/l fosfomycin, 15 mg/l metronidazole, 3 mg/l daptomycin) for the last three days of antibiotic treatment with the water changed daily. In the last 48 h, the copepods were starved and fed with 90 μl/l 0.6-μm copolymer beads (Sigma-Aldrich, St. Louis, MO) to remove their gut microbiome.

For DNA extraction of this initial sample, the DNeasy Blood & Tissue Kit (Catalog No. 69504, QIAGEN, Hilden, Germany) was used to obtain 48 μg of high molecular weight (HMW) genomic DNA. The extracted DNA was quantified by pulsed-field gel electrophoresis, NanoDrop Spectrophotometry (Catalog No. ND2000CLAPTOP, Thermo Fisher Scientific, Wilmington, DE) and Qubit 3.0 Fluorometry (Catalog No. Q33216, Thermo Fisher Scientific). The CLR library (PacBio, Menlo Park, CA) was constructed with 20 kb insert size using SMRTbell Template Prep Kit 1.0 (PacBio, Menlo Park, CA) following the manufacturer’s protocol. The DNA library was sequenced on four SMRT Cells using the PacBio Sequel II platform at Dovetail Genomics (Scotts Valley, CA) to generate 2.6 million reads (30.3 Gb, ∼ 60.6× coverage).

To validate the assembly quality and complement the sequencing coverage, an additional 1000 copepod individuals were collected. The cetyltrimethylammonium bromide (CTAB)-based phenol/chloroform/isoamylol DNA extraction approach, which we found to be superior for obtaining long-read DNA sequences for copepods, was performed to obtain 16 μg HMW genomic DNA (File S1). A PacBio HiFi CCS library was constructed with 10–20 kb insert sizes and sequenced on a SMRT Cell 8M using the PacBio Sequel II platform at Novogene (Sacramento, CA). A total of 0.59 million HiFi CCS reads (7.1 Gb, ∼ 14.2× coverage) were generated by calling consensus from subreads produced by multiple passes of the enzyme around a circularized template. Another 0.5 μg DNA sample was used to construct a 350 bp insert size library and sequenced on the Illumina HiSeq NovoSeq 6000 platform (San Diego, CA) at Novogene with 150 bp pair-end (PE) mode to generate 244.6 million reads (36.7 Gb, ∼ 73.4× coverage).

To assemble the genome to chromosome-level resolution, two Hi-C sequencing libraries were prepared following a protocol from Dovetail Genomics. The chromatin of 500 copepods was fixed with 2% formaldehyde for cross-linking in the nucleus and extracted afterward. DNA was digested with MboI restriction endonuclease with non-ligated DNA fragments removed. The ligated DNA was sheared to ∼ 350 bp followed by a standard Illumina library preparation protocol. The library was also sequenced on the Illumina HiSeq X Ten platform with 100 bp PE mode to generate 112 million 2 × 150 bp reads for the first library and 59 million 2 × 150 bp reads for the second library (for a total of 42.8 Gb, ∼ 85.6× coverage).

### Chromosome-level genome assembly of *E*. *carolleeae*

The genome size of *E*. *carolleeae* was estimated prior to genome assembly, using both our previous Illumina genome sequence data (NCBI BioProject PRJNA203087), generated in the i5K Arthropod Genome Pilot Project (NCBI BioProject PRJNA163973), and the newly generated Illumina sequence data in the present study. Genome size was estimated based on the *k*-mer distribution using Jellyfish [73] (parameters: count -m 21/25 -C -s 1G -F 2, histo -h 1,000,000). GenomeScope 2.0 [74] was used to estimate the genome size, heterozygosity, and proportion of repetitive sequences with *k* = 21 and *k* = 25.

The PacBio CLR data were first used solely to assemble the primary genome. The raw sequence reads were self-corrected using NextDenovo v2.3 (https://github.com/Nextomics; parameters: genome size = 500 m, seed_cutoff = 13 k, read_cutoff = 1 k, sort_options = -m 10g -t 2 -k 50, minimap2_options_raw = -t 8). The all-to-all alignment by minimap2 (parameters: -x ava-pb -t 8 -k17 -w17) and NextGraph in NextDenovo (parameters: -a 1) were used to generate the primary genome assembly. NextPolish was used to polish the genome assembly with both PacBio CLR reads and Illumina short reads. One round of long read polishing and three rounds of short read polishing (parameters: sgs_options = -max_depth 100) were performed successively to improve the assembly. To validate that the robustness of our assembly was not influenced by sequencing coverage, the corrected CLR data and HiFi CCS reads were combined and the primary genome was reassembled with the same parameters using NextDenovo v2.3. The N50 statistic (defined as the sequence length of the shortest contig at 50% of the total assembly length) was used to evaluate the genome contiguity of the primary assembly. The completeness of the genome assembly was assessed using BUSCO v5.2.2 at nucleotide level based on 1013 genes in the arthropoda_odb10 database [75]. These two assemblies, based on different datasets, showed very similar quality with respect to contiguity and completeness (shown in Table S17). This assembly (#1 in Table S17) with higher contig N50 was further used in the following analyses. Purge_Dups (https://github.com/dfguan/purge_dups) was applied to remove heterozygous duplicates in the genome assembly.

For chromosome scaffolding, Juicer [76] and 3D-DNA [77] were used to scaffold the genome assembly to the chromosome level. Juicebox was also used to generate the Hi-C contact map and manually correct the errors in scaffolding. Eleven scaffolds that were disconnected from the rest of the assembly were manually removed. The microbial sequences were identified and removed by searching the NCBI Nucleotide (NT) database.

### Karyotype of the *E*. *carolleeae* genome

Cytogenetic analysis of the *E*. *carolleeae* genome was performed by the UW Cytogenetic Services in the Wisconsin State Laboratory of Hygiene (WSLH). Live copepod samples were used to isolate cells in metaphase. Cells were swollen in a hypotonic solution (0.075 M KCl) for 20 min at 37°C, and then fixed three times in fresh Carnoy’s fixative. Cells were dropped onto slides and dried in a drying chamber. Slides were banded by the Giemsa banding technique and scanned to find cells with well isolated chromosomes.

### Chromosome number and genome size evolution across the Copepoda

To gain comparative insights into patterns of genome size and chromosome number evolution across the Copepoda, the available and published data were summarized for four copepod orders. These data integrated information from both genome assemblies present in NCBI Genome database and published cytophotometric and karyological investigations (Tables S2 and S3). The records for copepod species were also retrieved from the Animal Genome Size Database. The chromosome numbers were mapped onto a synthesis tree of the Copepoda that integrated 31 published phylogenies [78]. Statistical comparisons of chromosome number and genome size among the four copepod orders were performed with Kruskal–Wallis and pairwise Wilcoxon signed rank tests in R.

### Genome annotation of *E*. *carolleeae*

The MAKER v3.01 [79] pipeline was applied to annotate protein-coding regions of the *E. carolleeae* genome. Gene structure prediction was integrated using three strategies, *i.e.*, homology-based, transcriptome-based, and *ab initio* prediction. For homology evidence, the protein sequences of *D*. *melanogaster* (GCF_000001215.4), *Daphnia pulex* (GCF_021134715.1), *T*. *californicus* (GCF_007210705.1), *L*. *salmonis* (GCF_016086655.3), and *E*. *carolleeae* (GCF_000591075.1) in NCBI Reference Sequence (RefSeq) database release 216 were fed into MAKER. For transcriptomic evidence, a total of 52 transcriptomic datasets from *E*. *affinis* complex were used, including 46 that were sequenced in our previous gene expression study under various salinity treatments (NCBI BioProject PRJNA278152), three that were sequenced in our previous i5K Arthropod Genome Pilot Project (NCBI BioProject PRJNA275666), and two that were sequenced in this present study using samples from two other clades of the *E*. *affinis* species complex (Europe [*E*. *affinis* proper (Poppe, 1880)] and Gulf of Mexico) (File S1). These transcriptomic datasets were collected and reassembled based on our new reference genome, using HISAT v2.0.4 [80] and StringTie v2.2.1 [81]. Regarding *ab initio* gene prediction, the gene predictor SNAP was trained with the gene models predicted with the aforementioned evidence. The self-trained predictor GeneMark-ES was applied separately. Within MAKER, the genome was masked for repetitive regions, and protein homology and transcript sequences were aligned using Basic Local Alignment Search Tool (BLAST). Three iterative runs of MAKER were performed, with gene predictions from each run serving as training sets for the following run. Finally, MAKER evaluated the consistency across these different forms of evidence and generated a final set of gene models.

Functional annotation of gene models was performed by Protein BLAST (BLASTP) searches of the NCBI RefSeq and UniProtKB/Swiss-Prot databases of invertebrates using a separate self-established database with all gene sequences of *E*. *affinis* complex in RefSeq. The databases GO, Kyoto Encyclopedia of Genes and Genomes (KEGG), Clusters of Orthologous Genes (COG), and Evolutionary genealogy of genes: Non-supervised Orthologous Groups (EggNOG) were searched for functional annotation using eggNOG-mapper v2.1.9 [82]. The Pfam database in InterPro was also searched by HMMER v3.2.

To confirm the accuracy of the gene predictions, a read depth analysis was performed for all predicted genes. Our existing Illumina genome sequence data were mapped onto the genome assembly. The read depth for each predicted gene was calculated using BEDTools 2.30 [83].

To detect the relative ages of gene duplicates and evidence for ancient WGD, *Ks* frequency analysis was performed using the DupPipe pipeline (https://gitlab.com/barker-lab/EvoPipes). All protein-coding genes were translated to identify reading frames by comparing the GeneWise alignment to the best hit protein from the same homology protein sequences used in the genome annotation. Synonymous divergence (*Ks*) was estimated using phylogenetic analysis by maximum likelihood (PAML) with the F3 × 4 model [84].

RepeatMasker v4.07 (http://www.repeatmasker.org) was used to identify repetitive sequences in the genome based on searching in the Repbase v202101, Dfam v3.7, and a *de novo* repeat library built by RepeatModeler v1.0.8 (http://www.repeatmasker.org/RepeatModeler). Searches for LTR were also performed using the same databases. Unknown transposable elements were reclassified by DeepTE [85]. To compare the total number of transposable elements and the proportion of different types of transposable elements across copepod species, this annotation pipeline was applied to three additional copepod species with high-quality genomes in the NCBI Genome database (*i.e.*, *C*. *rogercresseyi*, *L*. *salmonis*, and *T*. *californicus*).

tRNAs were identified using tRNAscan-SE v2.0 [86] with default parameters. microRNAs (miRNAs) and small nuclear RNAs (snRNAs) were identified with Nucleotide BLAST (BLASTN) against the Rfam database v12.0, and ribosomal RNAs (rRNAs) were identified against other copepod rRNA sequences.

### Gene family expansions and contractions across the Arthropoda

Orthologous gene families in the *E*. *carolleeae* genome were identified by OrthoFinder v2.5.4 [87]. Protein sequences of 12 additional arthropod species with high-quality genomes, assembled with long-read sequences to the chromosome level, were downloaded from the GenBank database (Table S18). These arthropod genomes included two chelicerates (*Hyalomma asiaticum*, *Hylyphantes graminicola*), one barnacle (Thecostraca: *Pollicipes pollicipes*), three copepods (*C*. *rogercresseyi*, *L*. *salmonis*, *T*. *californicus*), four branchiopods (*D*. *pulex*, *Daphnia magna*, *Daphnia pulicaria*, *Daphnia sinensis*), and two hexapods (*D*. *melanogaster*, *Aphis gossypii*). Alternative splice variants were first filtered out for each gene and only the longest transcripts were kept. Proteins of our copepod *E*. *carolleeae* and other arthropod species were aligned using BLASTP (E-value < 1E−5).

A phylogeny was reconstructed using a maximum likelihood algorithm in RAxML v8.0.19 [88]. 100 bootstrap replicates were performed to assess statistical support for tree topology. MCMCTree from PAML v4.9 was used to estimate divergence time. Three confidence time intervals retrieved from the TimeTree v5 database were applied in MCMCTree as calibrations for the divergence time (shown as red dots in Figure 3). Computational Analysis of gene Family Evolution (CAFÉ) v5.0 [89] was used to analyze the expansions and contractions of gene families among taxa in the phylogenetic tree. For gene families exhibiting expansions and contractions in the *E*. *carolleeae* genome, GO and KEGG enrichment analyses were performed using TBtools v1.112 [90].

Syntenic relationships among three copepod species were analyzed using MCScan in JCVI (https://github.com/tanghaibao/jcvi). Only the highest quality available copepod genomes were used, namely, *E*. *carolleeae*, *T*. *californicus*, and *L*. *salmonis*, representing three different copepod orders, Calanoida, Harpacticoida, and Siphonostomatoida, respectively. Collinear gene blocks within the genomes were identified using the longest coding sequence of each gene.

### Genome-wide CpG_o/e_ values in the *E*. *carolleeae* genome

To assess the patterns of historical methylation within gene bodies, genome-wide CpG_o/e_ values for genes were determined across the *E*. *carolleeae* genome. The CpG_o/e_ value of each gene was computed as the observed frequency of CpG sites (*f*_CpG_) divided by the product of C and G frequencies (*f*_C_ and *f*_G_), *i.e.*, *f*_CpG_/*f*_C_*f_G_ in the coding sequence of each gene [38]. The density of CpG_o/e_ values for all genes was fitted and plotted in R. The distribution of CpG_o/e_ values per gene was also plotted based on the gene position on different chromosomes. To investigate the functional categories of genes with the highest and lowest CpG_o/e_ values, GO enrichment analysis was performed for the top 5% genes with the highest and lowest CpG_o/e_ values using TBtools.

### Localization of ion transport-related genes across the *E*. *carolleeae* genome

A total of 490 genes with ion (cation and anion) transporting function were mapped onto the four chromosomes based on our genome annotation (Figure 6A). In addition, 80 key ion transport-related genes that showed evolutionary shifts in gene expression and/or signatures of selection in prior studies [24] were manually annotated and mapped separately onto the chromosomes (Figure 6A). These ion transport-related genes are likely involved in hypothetical models of ion uptake (Figure 6B and C). These genes included paralogs of *NKA-α*, *NKA-β*, *NHA*, *NHE*, *NKCC*, *CA*, *AMT*, and *Rh*, subunits of *VHA*, and *SLC4* (including *AE*, *NBC*, and *NDCBE*) (Figure 6B and C, Figures S10 and S11).

Distances between adjacent ion transport-related genes were calculated, and deviation of the distance distribution of these genes from a uniform distribution was tested using the Kolmogorov–Smirnov test in R. In addition, deviation of the distance distribution of these ion transport-related genes from the distance distribution of the same number of functionally conserved genes was tested using the Chi-square goodness of fit test in R. For the functionally conserved genes, genes with the highest CpG_o/e_ values (identified in the Genome-wide CpG_o/e_ values in the *E*. *carolleeae* genome section) were used (Table S16). This set of genes was enriched in RNA processing and DNA binding related functions, which tend to be functionally conserved housekeeping genes.

## Supplementary Material

qzae066_Supplementary_Data

## Data Availability

The raw sequence data generated in this study have been deposited into the NCBI Sequence Read Archive (SRA) database (BioProject: PRJNA1075304). Genome assembly and gene annotations are available on figshare (https://doi.org/10.6084/m9.figshare.25386496.v1).

## CRediT author statement

**Zhenyong Du:** Formal analysis, Investigation, Data curation, Visualization, Writing – original draft, Writing – review & editing. **Gregory Gelembiuk:** Resources, Writing – review & editing. **Wynne Moss:** Resources, Writing – review & editing. **Andrew Tritt:** Resources, Writing – review & editing. **Carol Eunmi Lee:** Conceptualization, Project administration, Supervision, Funding acquisition, Writing – review & editing. The authors have read and approved the final manuscript.
