# Supplementary material for: The Genome Architecture of the Copepod Eurytemora carolleeae — the Highly Invasive Atlantic Clade of the Eurytemora affinis Species Complex
Source: Genomics Proteomics Bioinformatics. 2024 Sep 27;22(5):qzae066. doi: 10.1093/gpbjnl/qzae066 (PMC11706791; doi:10.1093/gpbjnl/qzae066)
Supplement: qzae066_Supplementary_Data [file qzae066_supplementary_data.zip › Supplementary material captions.docx]

**Supplementary materials**

**File S1 Supplementary methods**

**Figure S1 The k-mer coverage frequency plots for two different Illumina sequencing datasets and k-mer sizes for the copepod *Eurytemora carolleeae* (Atlantic clade of the *Eurytemora affinis* species complex)**

The top two graphs plotting the k-mer coverage are for the Illumina-seq data generated previously in Eyun et al., whereas the bottom two graphs are for the Illumina-seq data generated in the present study. Left plots: k = 21 and right plots: k = 25. The estimated genome size, proportion of non-repetitive unique sequences, homozygosity, and heterozygosity are shown in the plots. These results indicate that the *E*. *carolleeae* genome has a genome size of 509–540 Mb, with 0.5%–0.7% heterozygosity and 37%–43% repetitive sequence.

**Figure S2 Two different cells showing the karyotype of the copepod *Eurytemora carolleeae***

In each photograph, four pairs of chromosomes are clearly shown.

**Figure S3 Synteny among three chromosome-level copepod genomes**

Ecar, *Eurytemora carolleeae*; Tcal, *Tigriopus californicus*; Lsal, *Lepeophtheirus salmonis*. The source links for the genome sequences and annotations are available in Table S18.

**Figure S4 Distribution of Ks among paralogous genes within the *Eurytemora carolleeae* genome**

The Ks distribution was analyzed with all 20,262 protein-coding genes in the *E*. *carolleeae* genome. The Ks plot is truncated at Ks = 2 arbitrarily on the X-axis for ease of visualization. The high frequency of low Ks values indicates that the largest proportions of gene duplication events occurred quite recently (Ks = 0–0.04). Ks, synonymous substitutions per site.

**Figure S5 Proportions of repetitive sequence categories in four copepod genomes**

**A.** The proportions of each category of repetitive sequences as a percentage of each genome. Genome sizes are shown above the histogram for each species. **B.** The relative proportions of each category of repetitive sequences among all repetitive sequences. **C.** The proportions of each superfamily of retrotransposon and DNA transposon as a percentage of each genome. **D.** The relative proportions of each superfamily of retrotransposon and DNA transposon among all repetitive sequences. Only superfamilies representing more than 1% of each genome are included. Crog, *Caligus rogercresseyi*.

**Figure S6 Venn diagram showing the number of shared gene families among four pancrustacean species**

**Figure S7 KEGG enrichment of expanded genes in the copepod *Eurytemora carolleeae* genome**

These KEGG pathways were sorted by the *P* value (with higher *P* value downward). The complete list of enriched KEGG pathways is shown in Table S12. KEGG, Kyoto Encyclopedia of Genes and Genomes.

**Figure S8 Distributions of gene distance of all identified genes with ion transport function and key ion transport-related genes**

**A.** All 490 ion transport-related genes identified based on genome annotation (shown as vertical lines in Figure 6). **B.** The 80 key ion transport-related genes that showed evolutionary shifts in gene expression and/or signatures of selection in prior studies (shown as vertical lines with colored dots in Figure 6). Gene distances were calculated between adjacent target genes on the same chromosomes. Red dashed lines indicate the hypothetical mean distance between genes, if the genes had a uniform distribution across the genome. The set of all 490 identified genes with ion transport function (*P* = 1.1E−25) as well as the set of 80 key ion transport-related genes (*P* = 3.2E−30) on the chromosomes deviated significantly from a uniform distribution and tended to be more clustered than expected.

**Figure S9 Distributions of gene distance for all genes identified with ion transporting function and key ion transport-related genes, relative to functionally conserved genes**

**A.** All 490 ion transport-related genes identified from genome annotation (Figure 6, vertical lines). **B.** The 80 key ion transport-related genes that showed evolutionary shifts in gene expression and/or signatures of selection in prior studies (Figure 6, vertical lines with colored dots). Gene distances were calculated between adjacent target genes on the same chromosomes. The functionally conserved genes (blue bars) refer to genes with the highest CpG_o/e_ values identified in the *E*. *carolleeae* genome (Table S14) and are enriched with RNA processing and DNA binding related functions, which tend to be functionally conserved housekeeping genes. High frequencies of closely spaced genes were found for the 490 genes identified with ion transporting function (distance < 1E5 bp) as well as for the 80 key ion transport-related genes (distance < 1E6 bp). The distributions of gene distance differed significantly from those of functionally conserved genes for the 490 genes with ion transporting function (Chi-square goodness of fit test, *P* = 1.3E−9) as well as for the 80 key ion transport-related genes (Chi-square goodness of fit test, *P* = 3.9E−7).

**Figure S10 Localization of ion transport-related genes onto the four chromosomes of *Eurytemora carolleeae***

A total of 490 ion transport-related genes were identified from genome annotation (vertical lines). Among them, 80 were key ion transport-related genes (vertical lines with colored dots) that showed evolutionary shifts in gene expression and/or signatures of selection between saline and freshwater populations in our previous studies. See Posavi et al. for a more detailed list of ion transport-related genes that showed evolutionary shifts in gene expression, and Stern and Lee, Stern et al., and Diaz et al. for ion transport-related genes that showed signatures of natural selection in response to salinity change.

**Figure S11 Localization of ion transport-related genes on chromosome 3**

The magnified portion showing the region near the centromere of this chromosome.

**Figure S12 Hi-C contact map for three chromosomes of the *Eurytemora carolleeae* genome**

The stars indicate the positions of the centromeres.

**Table S1 Comparison of published copepod genome assemblies**

**Table S2 Karyotype of four copepod orders**

**Table S3 Genome sizes of four copepod orders**

**Table S4 Statistics of predicted protein-coding gene in the Eurytemora carolleeae genome**

**Table S5 Functional annotation of protein-coding genes in the *Eurytemora carolleeae* genome**

**Table S6 Repetitive sequence in the *Eurytemora carolleeae* genome**

**Table S7 Non-coding RNA genes identified in the *Eurytemora carolleeae* genome**

**Table S8 Single copy ortholog genes in the *Eurytemora carolleeae* genome used for the phylogenetic reconstruction**

**Table S9 Expanded genes in the *Eurytemora carolleeae* genome**

**Table S10 Contracted genes in the *Eurytemora carolleeae* genome**

**Table S11 GO enrichment of expanded genes in the *Eurytemora carolleeae* genome**

**Table S12 KEGG enrichment of expanded genes in the *Eurytemora carolleeae* genome**

**Table S13 Key ion transport-related genes in the *Eurytemora carolleeae* genome**

**Table S14 GO enrichment of genes with the 5% lowest CpG_o/e_ values in the *Eurytemora carolleeae* genome**

**Table S15 GO enrichment of genes with the 5% highest CpG_o/e_ values in the *Eurytemora carolleeae* genome**

**Table S16 Functionally conserved genes used in the comparison of distribution with ion transport-related genes**

**Table S17 Comparison of genome assemblies for *Eurytemora carolleeae* using different datasets and methods**

**Table S18 Arthropod genome assemblies used in the comparative genomic analyses**
