# Supplementary material for: The Genome Architecture of the Copepod Eurytemora carolleeae — the Highly Invasive Atlantic Clade of the Eurytemora affinis Species Complex
Source: Genomics Proteomics Bioinformatics. 2024 Sep 27;22(5):qzae066. doi: 10.1093/gpbjnl/qzae066 (PMC11706791; doi:10.1093/gpbjnl/qzae066)
Supplement: qzae066_Supplementary_Data [file qzae066_supplementary_data.zip › File S1.docx]

**File S1 Supplementary methods**

**Cetyltrimethylammonium bromide extraction protocol**

The copepods were homogenized in 800 μl cetyltrimethylammonium bromide (CTAB) solution (2% w/v, Catalog No. C2190, Teknova, Hollister, CA) with 30 μl proteinase K (Catalog No. 25530049, Invitrogen, Waltham, MA), and incubated in a water bath at 56°C overnight. The homogenized sample was treated with RNase (Catalog No. AM2271, Invitrogen, Waltham, MA) and incubated at 37°C for 30 min. 800 μl Tris-phenol: chloroform: isoamyl alcohol 25:24:1 (Catalog No. P2069, Sigma-Aldrich, St. Louis, MO) was added and centrifuged at 12000 r/min for 15 min. The upper phase was transferred into a new microtube with 800 μl chloroform: isoamyl alcohol 24:1 (Catalog No. C0549, Sigma-Aldrich, St. Louis, MO) added and centrifuged at 12000 r/min for 15 min. The upper phase was transferred into a new microtube, followed by 600 μl isopropanol (Catalog No. W292907, Sigma-Aldrich, St. Louis, MO) precipitation at –20°C for 2 h and centrifuged at 12000 r/min for 30 min. The pellet was rinsed twice with 70% ethanol, air-dried, and resuspended overnight at room temperature in Tris-EDTA buffer (Catalogue No. AM9849, Invitrogen, Waltham, MA).

**Transcriptome sequencing**

To supplement the genome annotation, two transcriptomes were sequenced using samples from two other clades of *Eurytemora affinis* complex, specifically inbred lines from the Gulf clade and Europe clade (Lee 1999). The Gulf clade copepods were collected from Blue Hammock Bayou, Louisiana, USA, and then inbred for 20 generations by full-sib mating. The Europe clade copepods (*E*. *affinis* proper) (Poppe 1880) were collected in Stockholm, Sweden, and then inbred for 10 generations through full-sib mating. All these copepods were maintained in the laboratory following the methods and conditions described in the Materials and methods section of the main text. Total RNA was isolated using TRIzol reagent (Catalog No. 15596026, Invitrogen, Waltham, MA) using ~ 100 pooled individuals according to the manufacturer’s instructions and subjected to quality control using NanoDrop Spectrophotometer (Catalog No. ND2000CLAPTOP, Thermo Fisher, Wilmington, DE), gel electrophoresis, and Agilent 2100 Bioanalyzer (Catalog No. G2938A, Santa Clara, CA). RNA samples were used for library preparation and sequenced on the Illumina HiSeq X Ten platform with the 100 bp pair-end mode. 377.9 million reads were generated for the Gulf clade and 372.4 million reads for the Europe clade.
