# Supplementary figures and images for: The Genome Architecture of the Copepod Eurytemora carolleeae — the Highly Invasive Atlantic Clade of the Eurytemora affinis Species Complex
Source: Genomics Proteomics Bioinformatics. 2024 Sep 27;22(5):qzae066. doi: 10.1093/gpbjnl/qzae066 (PMC11706791; doi:10.1093/gpbjnl/qzae066)

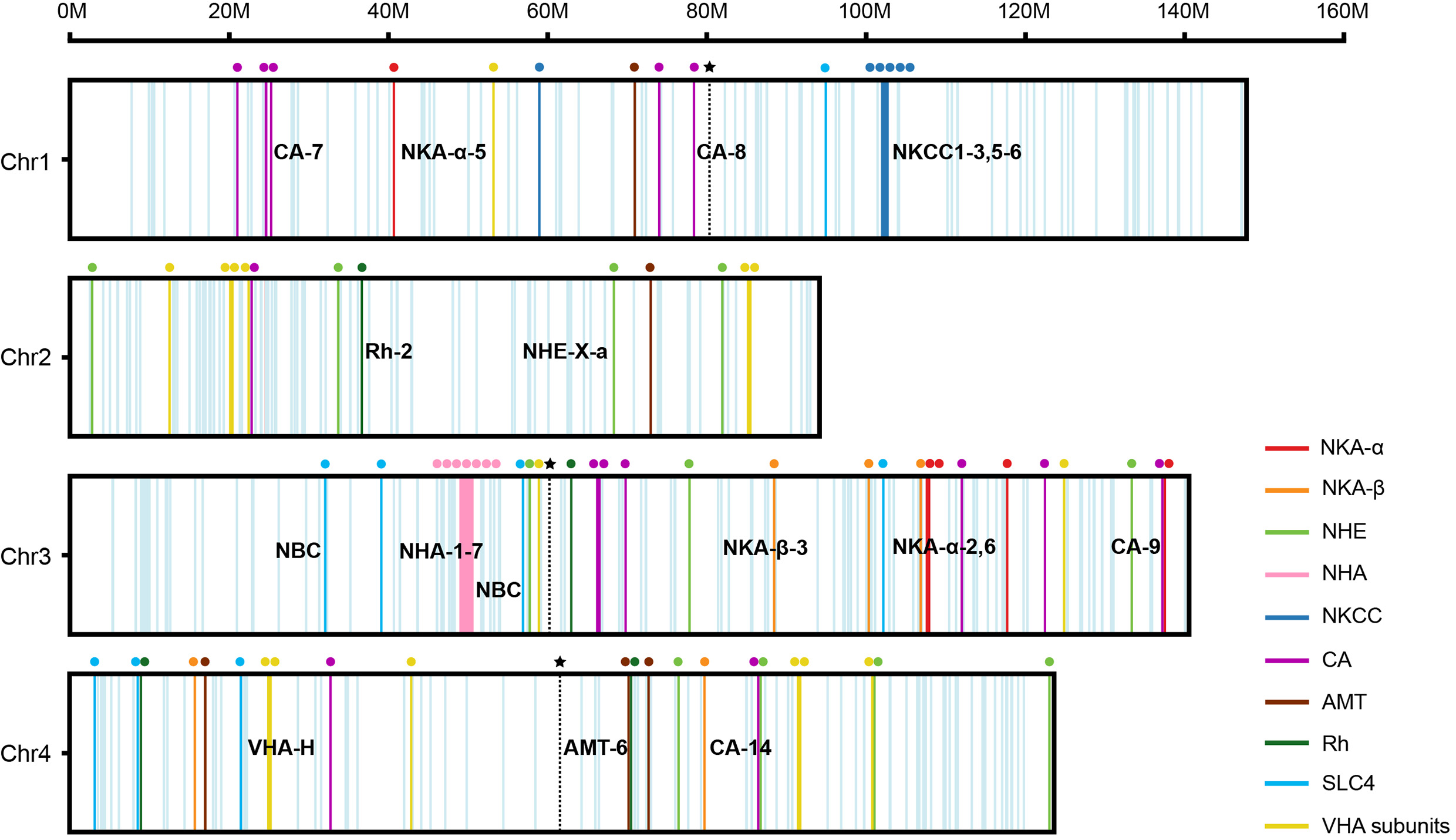

Supplement: qzae066_Supplementary_Data [file qzae066_supplementary_data.zip › Figure S10.jpg]

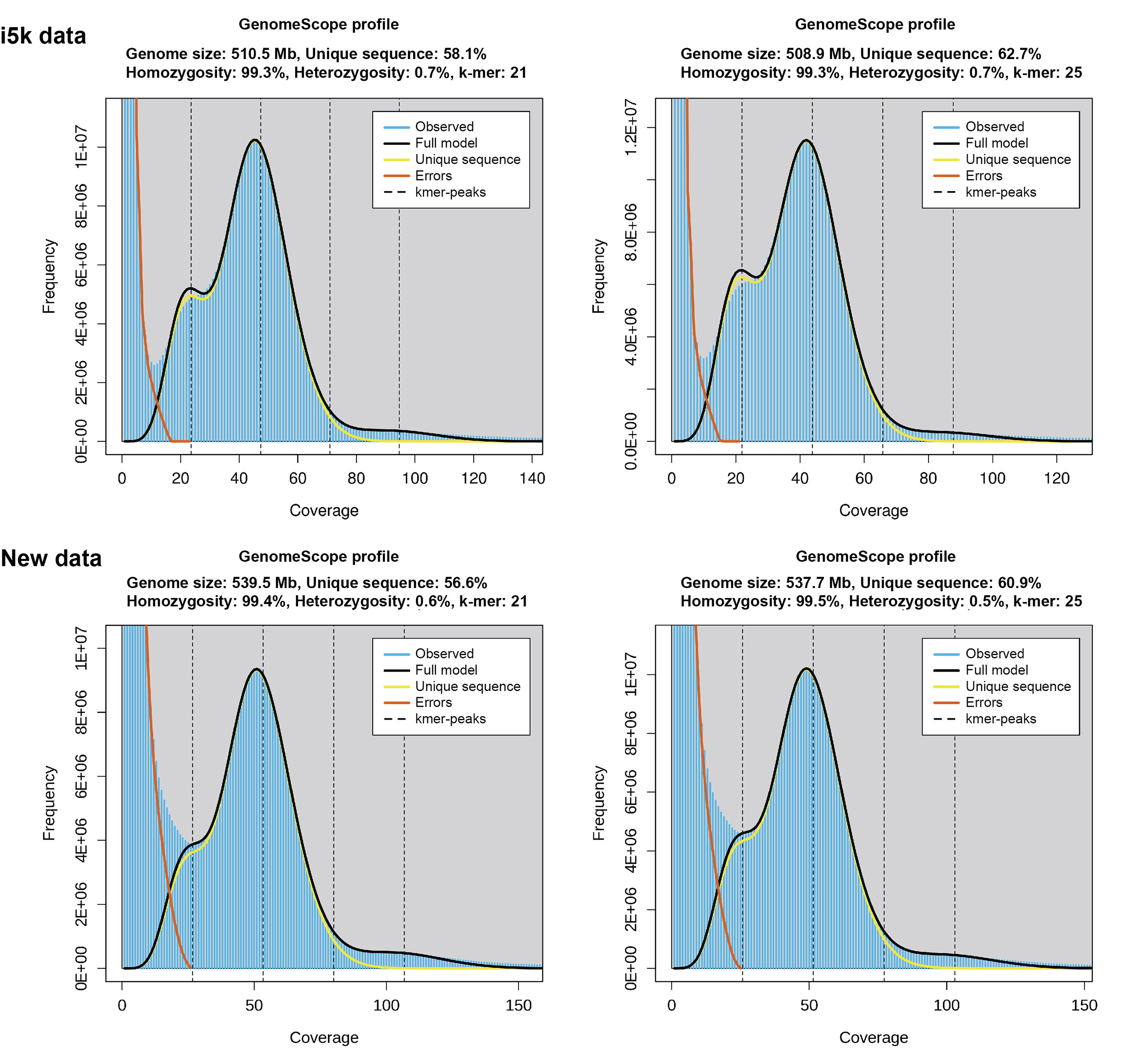

Supplement: qzae066_Supplementary_Data [file qzae066_supplementary_data.zip › Figure S1.jpg]

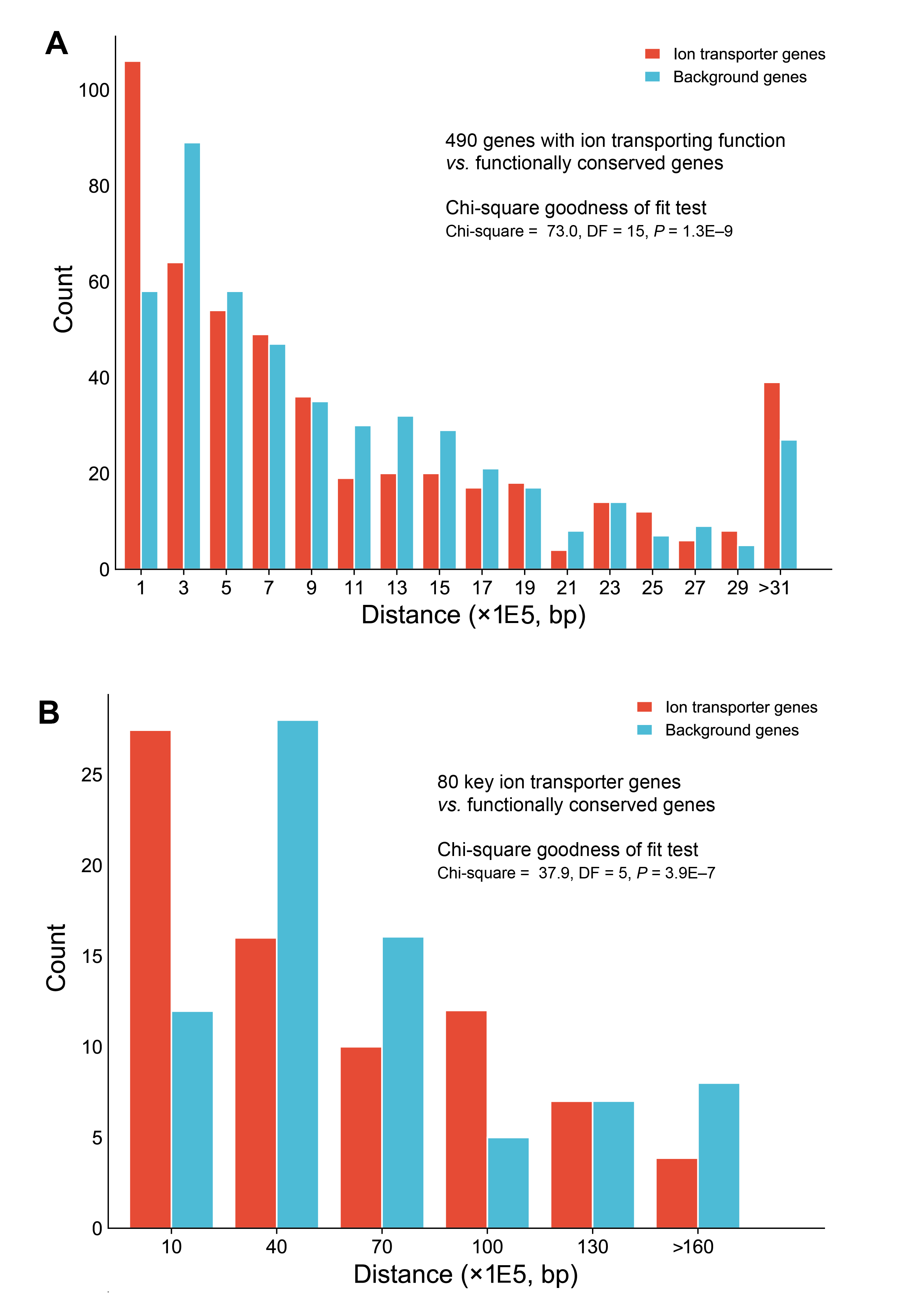

Supplement: qzae066_Supplementary_Data [file qzae066_supplementary_data.zip › Figure S9.jpg]

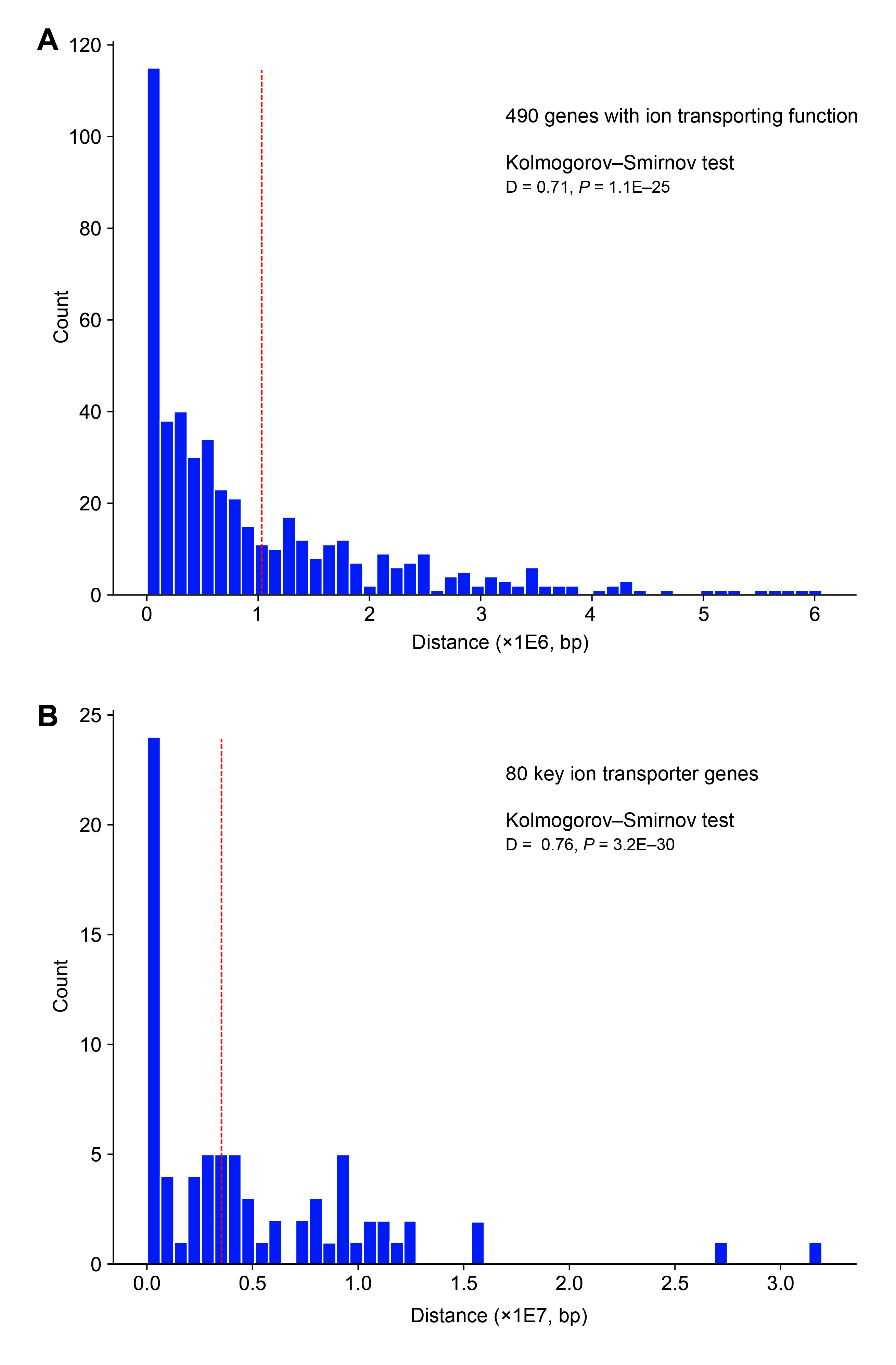

Supplement: qzae066_Supplementary_Data [file qzae066_supplementary_data.zip › Figure S8.jpg]

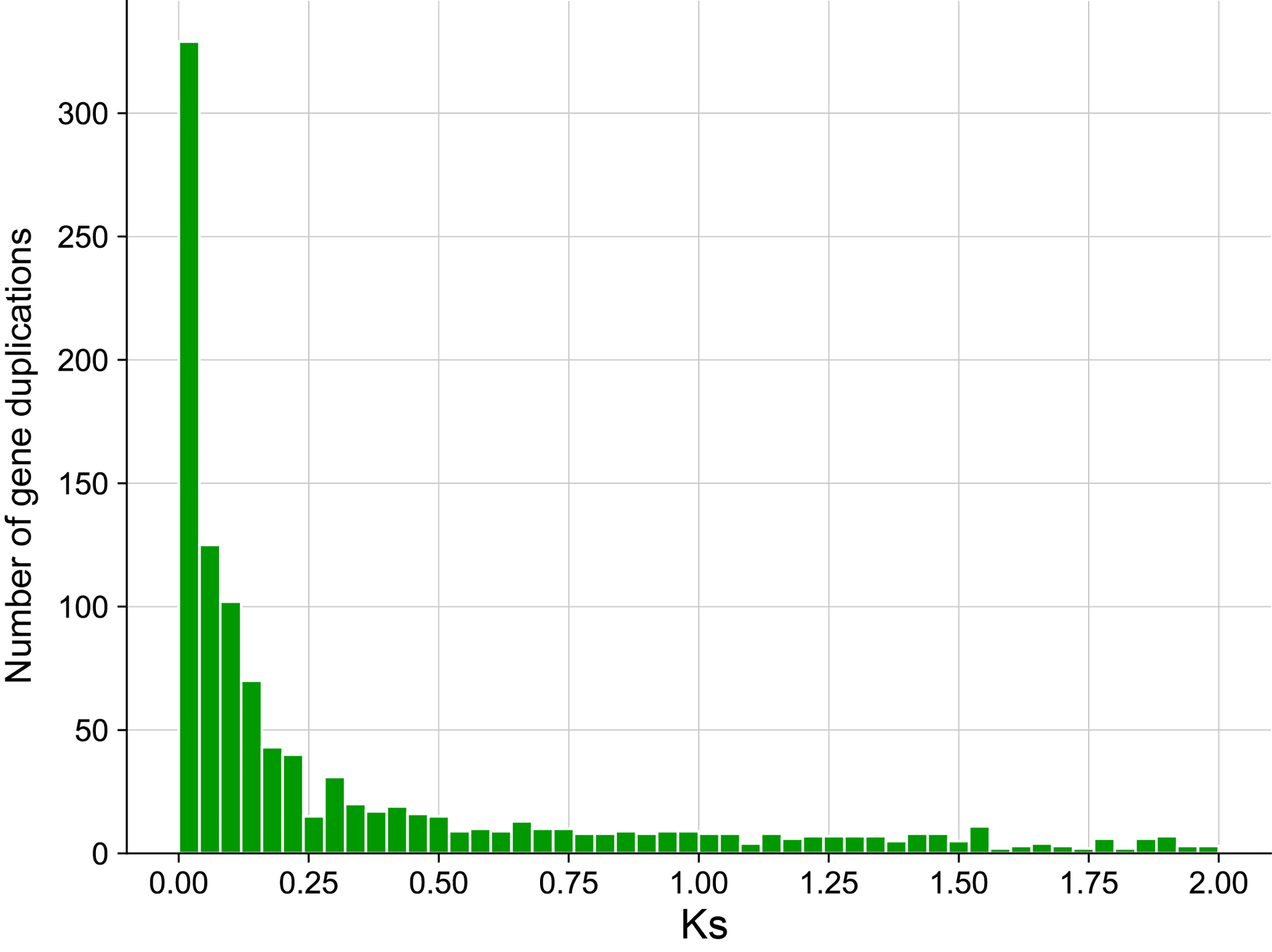

Supplement: qzae066_Supplementary_Data [file qzae066_supplementary_data.zip › Figure S4.jpg]

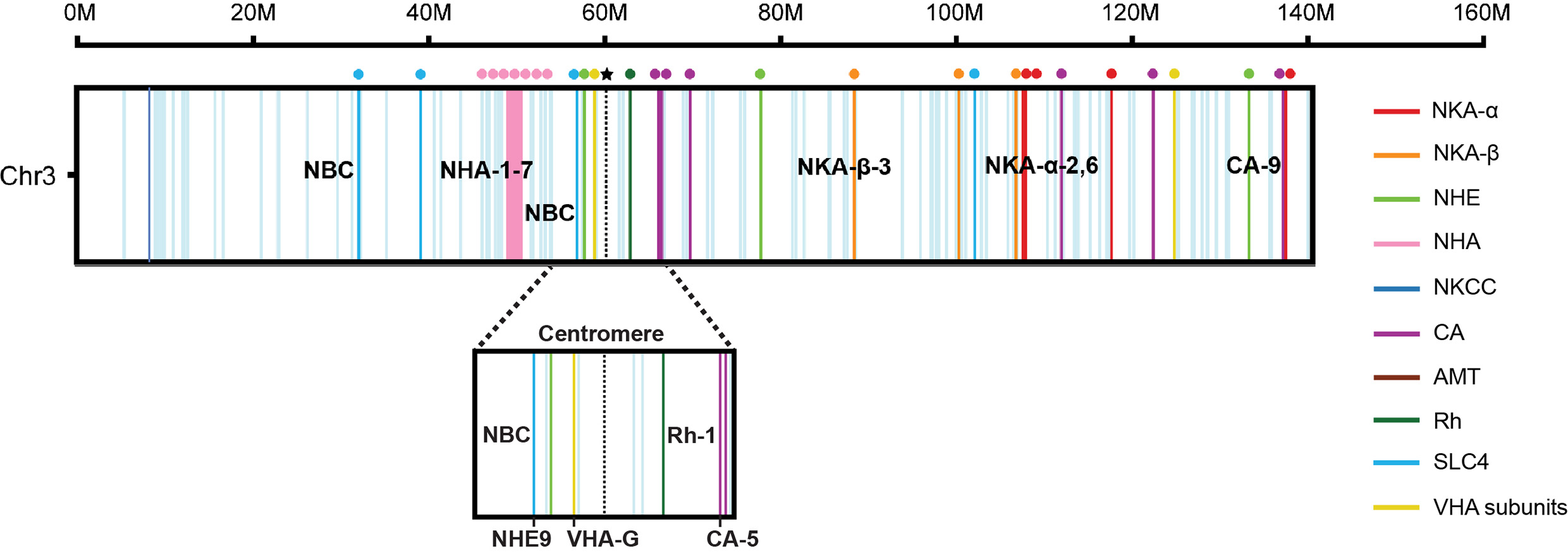

Supplement: qzae066_Supplementary_Data [file qzae066_supplementary_data.zip › Figure S11.jpg]

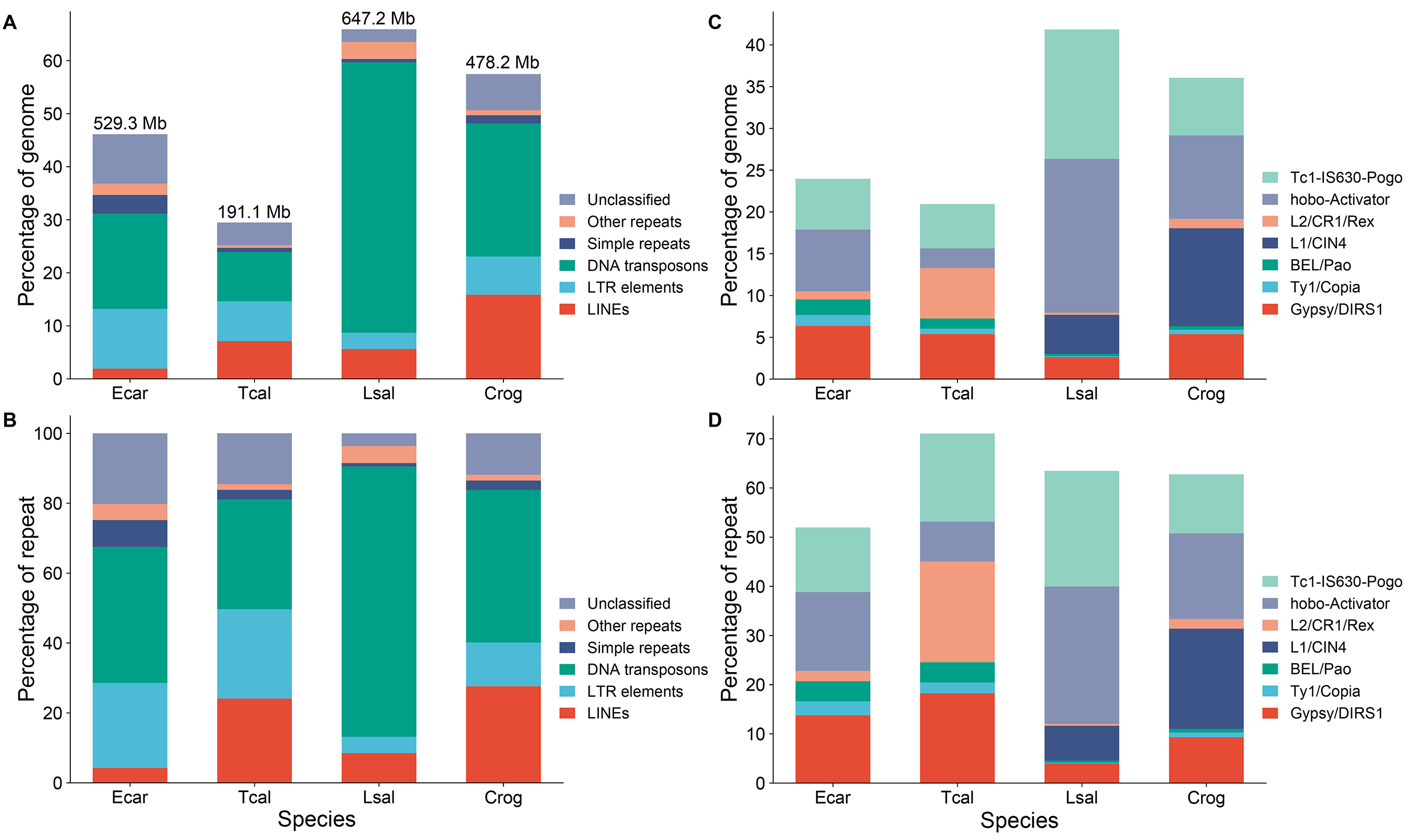

Supplement: qzae066_Supplementary_Data [file qzae066_supplementary_data.zip › Figure S5.jpg]

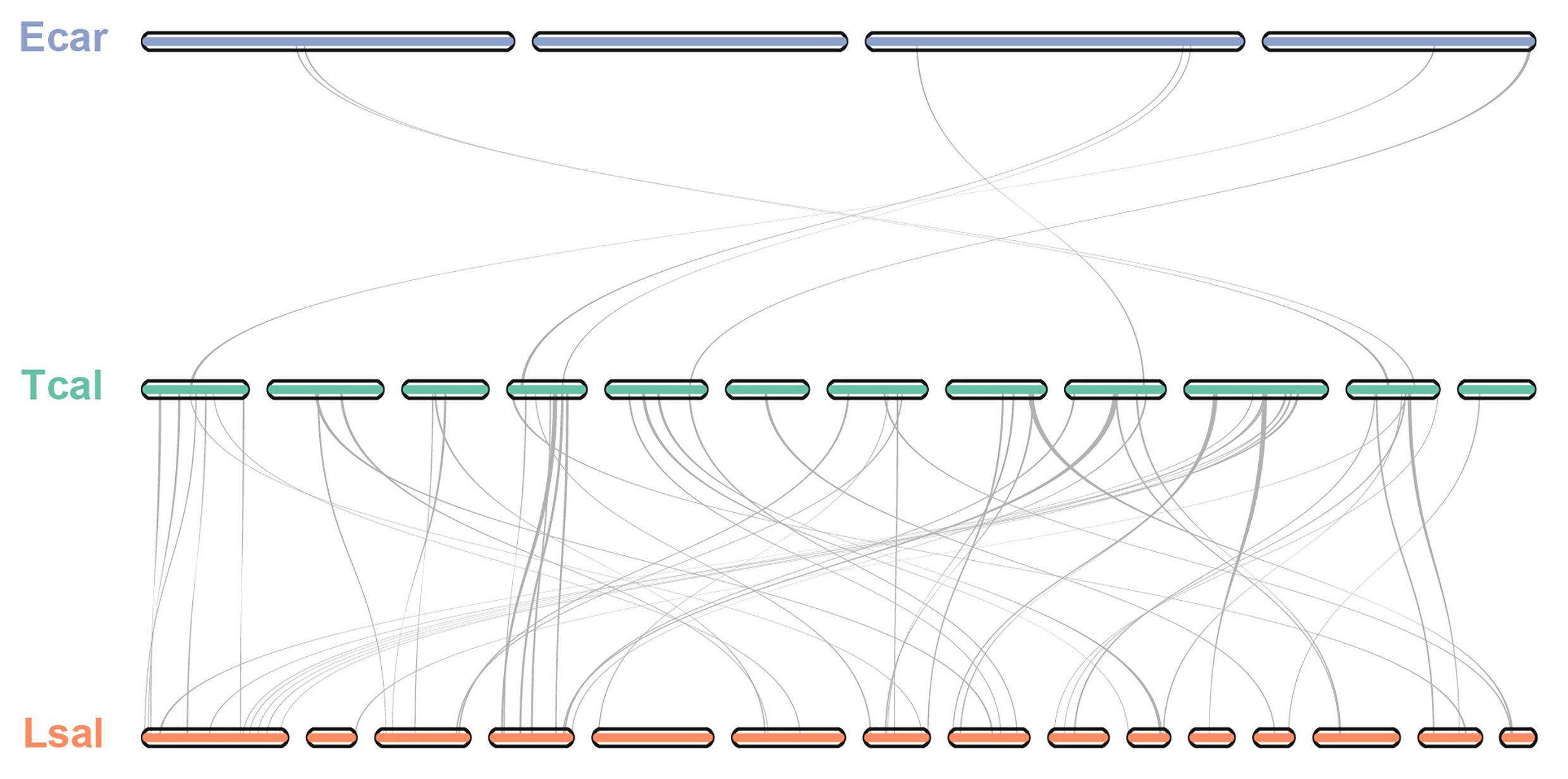

Supplement: qzae066_Supplementary_Data [file qzae066_supplementary_data.zip › Figure S3.jpg]

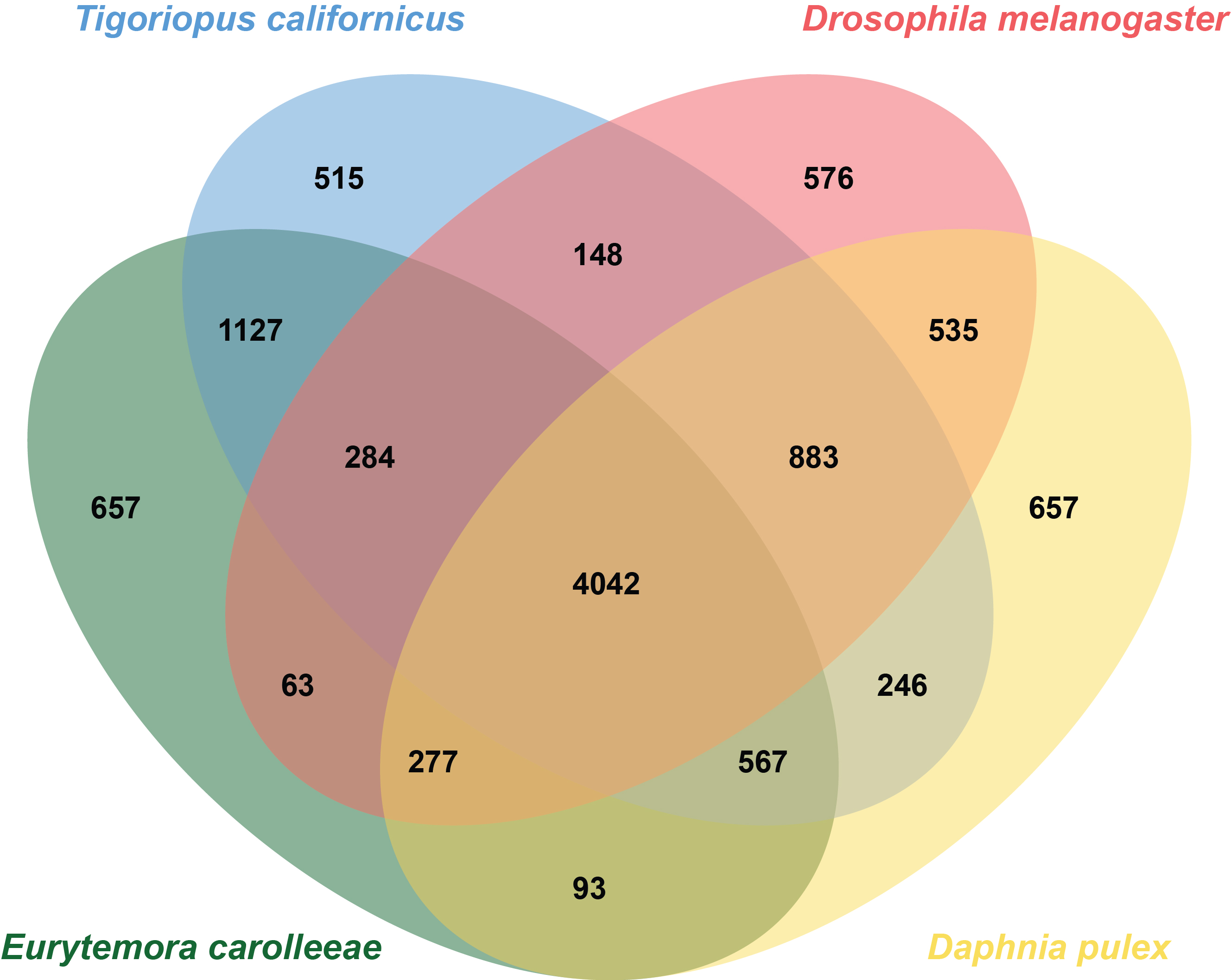

Supplement: qzae066_Supplementary_Data [file qzae066_supplementary_data.zip › Figure S6.jpg]

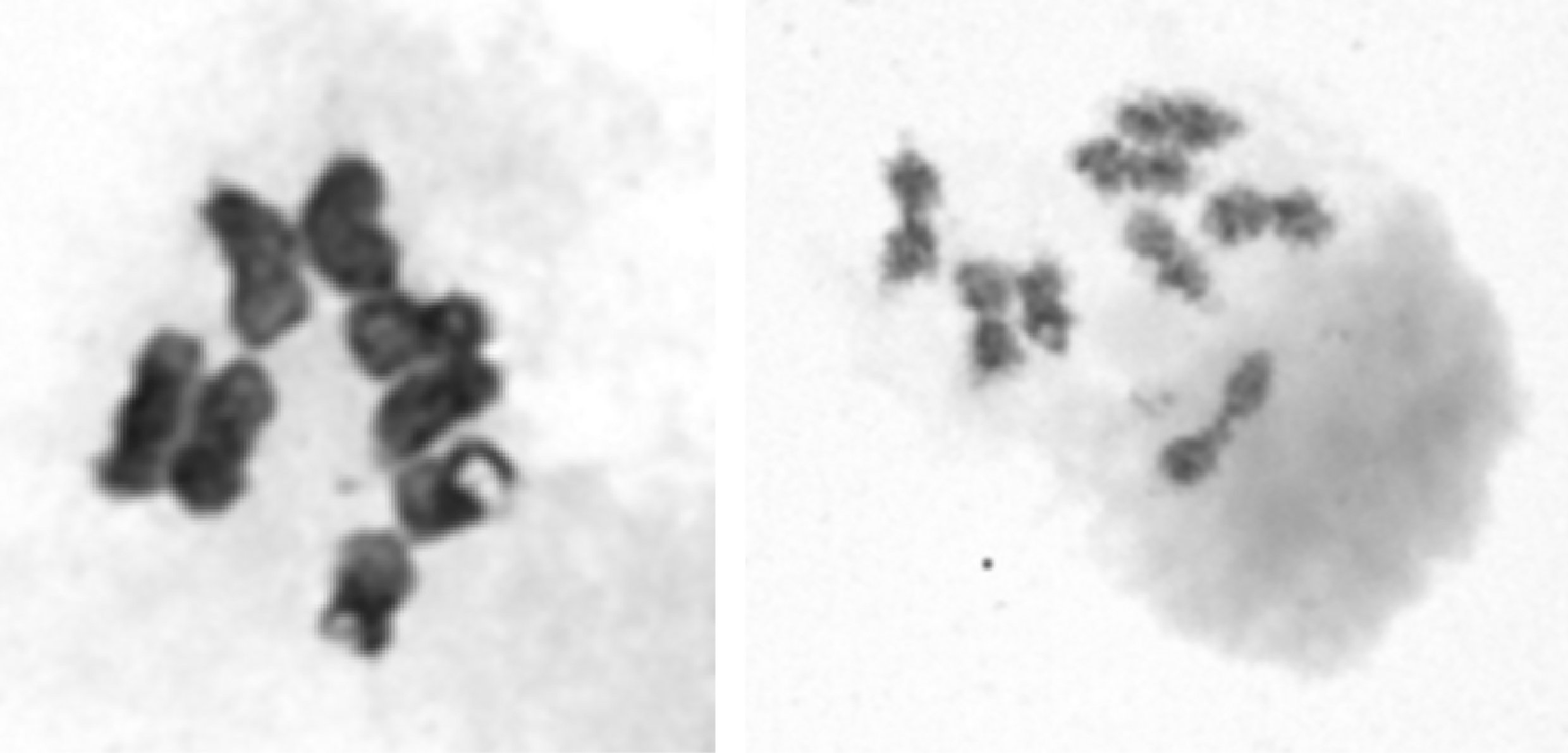

Supplement: qzae066_Supplementary_Data [file qzae066_supplementary_data.zip › Figure S2.jpg]

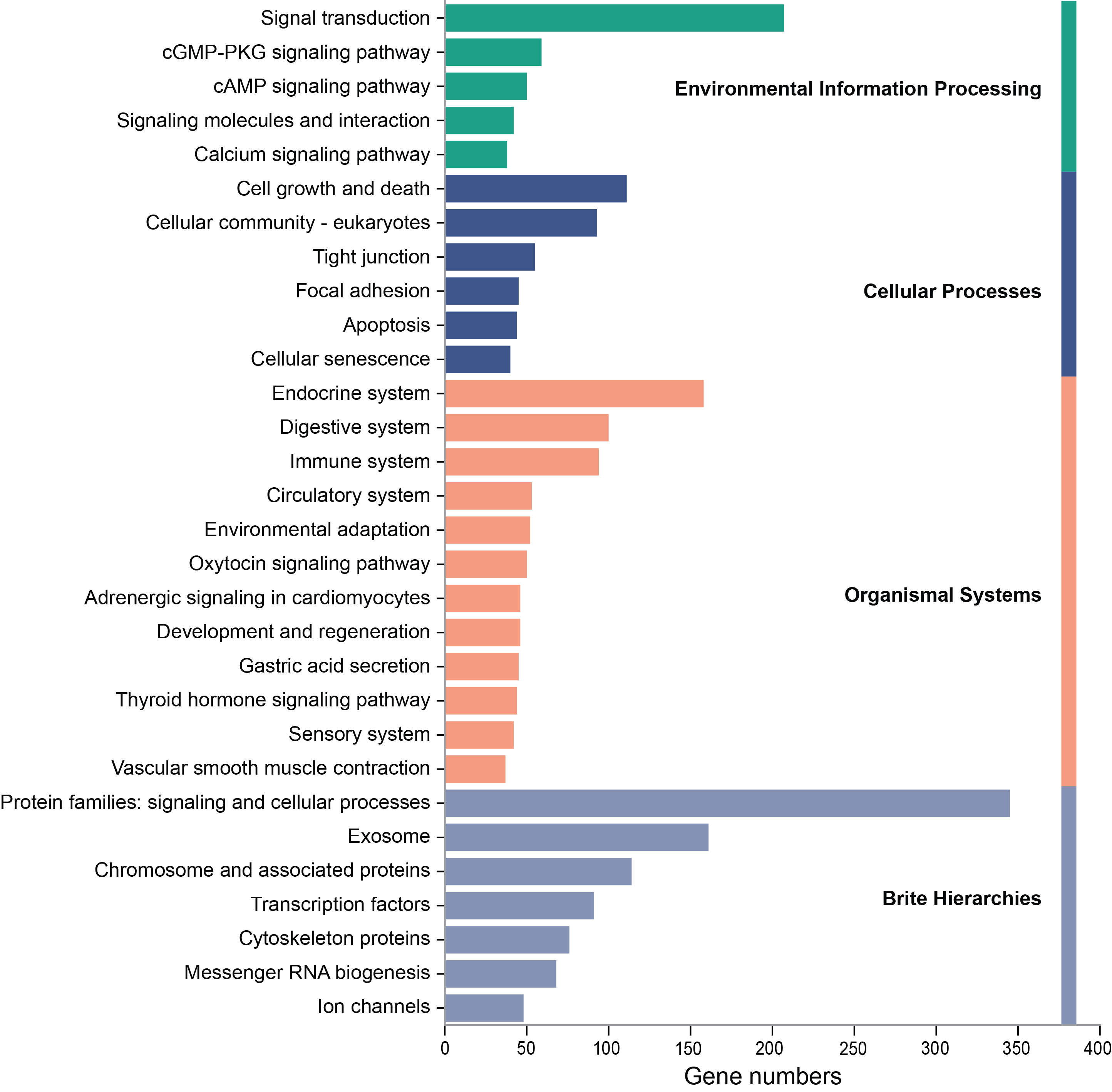

Supplement: qzae066_Supplementary_Data [file qzae066_supplementary_data.zip › Figure S7.jpg]

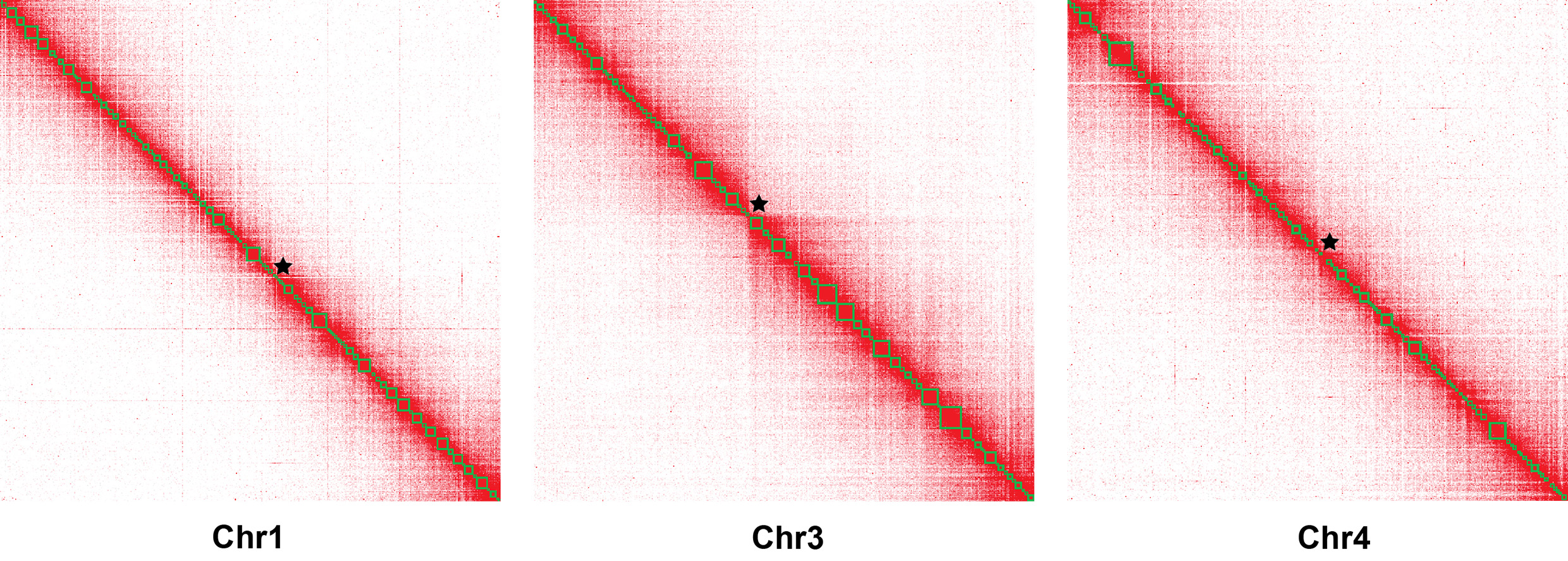

Supplement: qzae066_Supplementary_Data [file qzae066_supplementary_data.zip › Figure S12.jpg]
